# Ubiquitin ligase Nedd4 regulates the abundance and toxicity of mutant huntingtin

**DOI:** 10.1172/jci.insight.181013

**Published:** 2026-02-23

**Authors:** Hyunkyung Jeong, Yiyang Qin, Fangke Xu, Katarina Trajkovic, Myung Jong Kim, Nicolas Marotta, Kana Hamada, Ravi Allada, Su Yang, Dimitri Krainc

**Affiliations:** 1 Department of Neurology, Northwestern University Feinberg School of Medicine, Chicago, Illinois, USA.; 2 Guangdong Provincial Key Laboratory of Non-human Primate Research, Guangdong-Hong Kong-Macau Institute of CNS Regeneration, Jinan University, Guangzhou, China.; 3 Department of Neurobiology, Northwestern University, Evanston, Illinois, USA.

**Keywords:** Cell biology, Neuroscience, Neurodegeneration

## Abstract

Huntington’s disease (HD) is a neurodegenerative disorder caused by the expansion of CAG repeats in the gene encoding huntingtin. Since accumulation of mutant huntingtin (mHtt) leads to dysfunction of numerous cellular pathways and toxicity, reducing levels of the mutant protein represents a key therapeutic objective in HD. We found that ubiquitination of mHtt by E3 ubiquitin ligase Nedd4 promotes clearance of the mutant protein. Knockdown of Nedd4 increased toxicity of mHtt in mouse primary neurons and in a fly model of HD, suggesting the protective role of Nedd4. Importantly, levels of Nedd4 were decreased in mHtt-expressing neurons through impaired mTORC1 activity, suggesting a feedback loop of mHtt accumulation and Nedd4 reduction that leads to accumulation and, ultimately, toxicity of mHtt. These findings suggest that restoring Nedd4 activity may offer a novel therapeutic opportunity for HD.

## Abstract 2

<strong>Ubiquitin ligase Nedd4 regulates the abundance and toxicity of mutant huntingtin.</strong>

## Introduction

Huntington’s disease (HD) is a progressive neurodegenerative disorder characterized by motor, cognitive, and psychiatric symptoms. Typically, patients develop chorea, dystonia, impaired posture, balance, and gait, accompanied with cognitive decline and depression. HD is a monogenic, autosomal dominant disorder caused by the expansion of a CAG trinucleotide repeat in the huntingtin gene, which results in the extended polyglutamine (polyQ) sequence near the N-terminus of mutant protein huntingtin (mHtt). The disease is caused by a toxic gain-of-function of mHtt, which initially leads to dysfunction and degeneration of striatal medium spiny neurons but eventually affects other brain areas as well.

Huntingtin (Htt) is a large protein with a molecular weight of 348 kDa that undergoes proteolytic cleavage by caspases, calpains, and other proteases, and the resulting N-terminal fragments may contribute to toxicity in mutation carriers. Mutated, expanded polyQ versions of Htt and its fragments tend to form aggregates that have been associated with toxicity, although some studies claim that the soluble versions of the protein may be more harmful (1, 2). Nevertheless, neuropathology and motor dysfunction can be reversed by blocking the expression of mHtt in a conditional mouse model of HD (3), suggesting that reducing the levels of mHtt in affected individuals might have therapeutic effects. Moreover, application of antisense oligonucleotides with a goal of reducing the expression of mHtt has been advanced to clinical trials (4).

Accumulation of mHtt is mostly caused by its impaired clearance in affected cells. The stability and the clearance of mHtt is regulated by numerous posttranslational modifications (PTMs), such as phosphorylation, acetylation, palmitoylation, SUMOylation, and ubiquitination. For instance, acetylation of mHtt at K444 promotes its targeting to autophagosomes (5), and SUMOylation at K6, K9, and K15 has been linked to increased stability of mHtt, whereas ubiquitination at these residues promotes degradation of mHtt (6). The switch between ubiquitination and SUMOylation at these residues is determined by mHtt phosphorylation at S13 and S16 (7, 8). Identifying PTMs that would efficiently reduce mHtt is, thus, a potential tool to ameliorate the toxicity of the protein and the resulting neuropathology.

Nedd4-1 (neural precursor cell expressed, developmentally downregulated 4-1; Nedd4 hereafter) is a Homologous to the E6-AP C-terminus–type (HECT-type) E3 ubiquitin (Ub) ligase that mediates both monoubiquitination (9) and polyubiquitination of proteins (10, 11) targeting them for lysosomal (12, 13) or proteasomal degradation (14–17). Furthermore, Nedd4 has a broader role in protein degradation through regulation of autophagy (18, 19). For instance, it has been shown that Nedd4 interacts with autophagy-related LC3 (19, 20) as well as that it ubiquitinates p62 and modulates the process of p62-regulated inclusion body autophagy (10, 20). Interestingly, Nedd4 has been implicated in neuroprotection as it mediates lysosomal degradation of alpha-synuclein (12, 21) and restores ER-to-Golgi trafficking perturbed by alpha-synuclein (22, 23).

In this study, we identified an interplay between mHtt and Nedd4 whereby Nedd4 promotes monoubiquitination and degradation of mHtt, and mHtt in turn leads to downregulation of Nedd4. Such a feedback loop leads to decreased Nedd4-mediated ubiquitination and consequently further accumulation of mHtt. Maintaining the levels and/or activity of Nedd4 could thus interrupt this vicious cycle through reducing the levels of mHtt.

## Results

### Nedd4 promotes degradation of mHtt.

**Figure 1:**
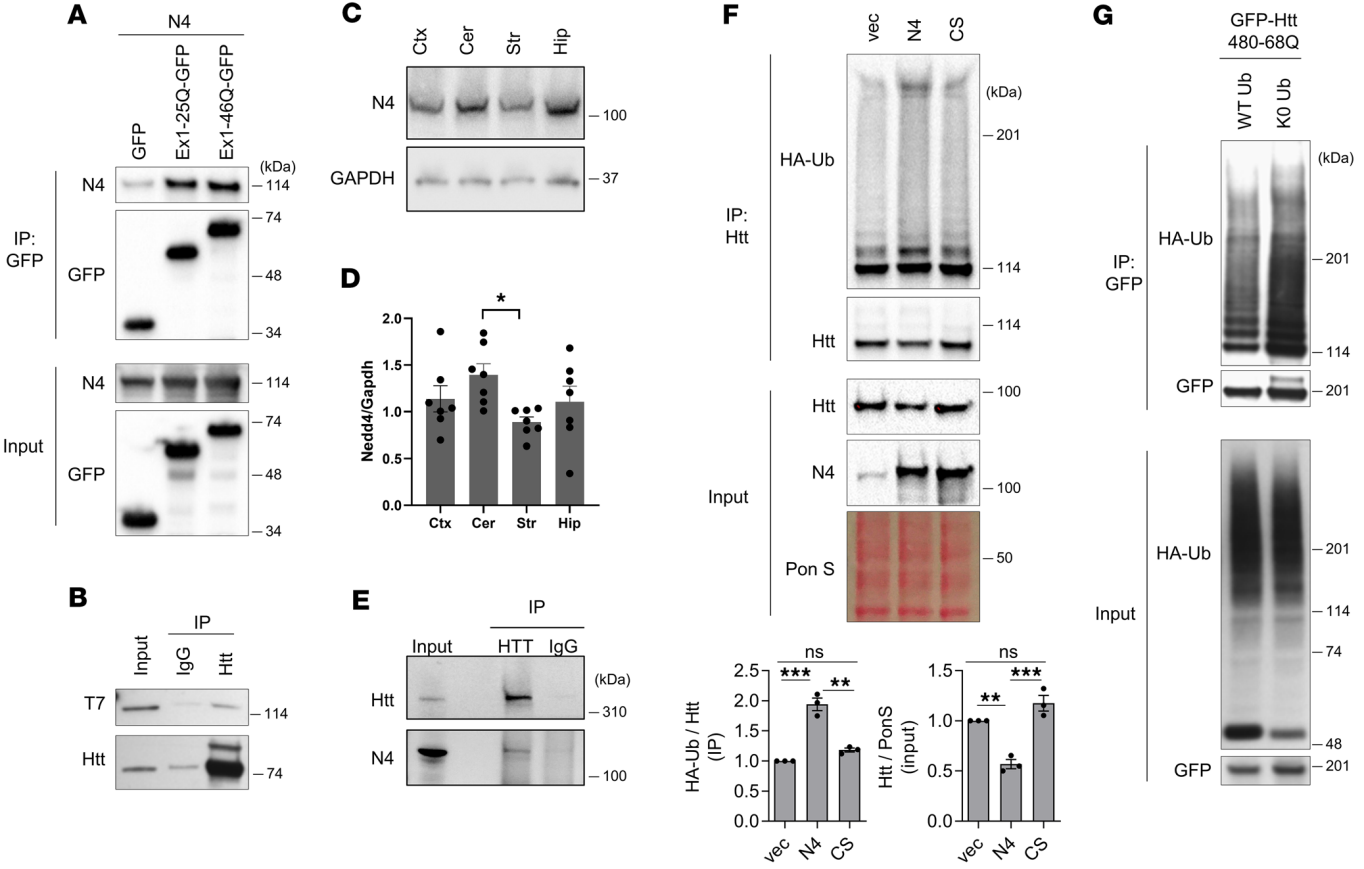
Nedd4 is an E3 ubiquitin ligase for Htt. (**A**) Interaction between Nedd4 and Htt Exon1-GFP. 293FT cells were transfected with Nedd4 together with GFP, Htt Exon1-25Q-GFP, or Htt Exon1-46Q-GFP. Cells were harvested 48 hours after transfection, and co-IP was performed followed by Western blot analysis. (**B**) Interaction of Nedd4 with Htt480-68Q. N2a cells were transfected with Nedd4 and Htt480-68Q and collected 26 hours after transfection. Co-IP was performed followed by Western blot analysis. T7 antibody was used to detect T7-tagged Nedd4. (**C**) Immunoblot analysis of Nedd4 expression in mouse brain regions: cortex (Ctx), cerebellum (Cer), striatum (Str), and hippocampus (Hip). Nedd4 was detected using an anti-Nedd4 antibody (Proteintech, 21698-1-AP), and Gapdh served as a loading control. (**D**) Quantification of relative Nedd4 expression normalized to Gapdh (*n* = 7; one-way ANOVA with Tukey’s post hoc test; Cer vs. Str, *P* = 0.0461). (**E**) Endogenous interaction between Htt and Nedd4 in mouse cortex. Co-IP was performed using cortical lysates, with Htt immunoprecipitated using an Htt antibody (EPR5526). Nedd4 was detected in the pulldown. IgG served as a negative control. (**F**) Nedd4 promotes Htt ubiquitination in a ligase activity–dependent manner. N2a cells were transfected with Htt571-72Q and HA-ubiquitin together with vector control, Nedd4, or catalytically inactive Nedd4-CS. Cells were harvested 50 hours after transfection, and denaturing IP was followed by Western blotting. *n* = 3; one-way ANOVA with Tukey’s multiple-comparison test (*P < 0.01; **P < 0.001; NS, not significant). (**G**) Htt undergoes monoubiquitination at multiple lysine residues. N2a cells were transfected with GFP-Htt480-68Q and either WT HA-ubiquitin or lysine-free HA-K0 ubiquitin. Denaturing IP and Western blot analysis were performed 25 hours after transfection. N4, Nedd4; Ub, ubiquitin; vec, vector; CS, Nedd4 CS; Pon S, Ponceau S.

Nedd4 is an E3 Ub ligase that harbors 4 WW domains— 40 amino acid-long protein modules containing two conserved tryptophans (W) which are spaced 20-22 amino acids apart. WW domains have high affinity for proline-rich domains (24–26), such as the ones found near the N-terminus of Htt. Therefore, we examined the interaction of Nedd4 and Htt by coimmunoprecipitation (co-IP) and found that Nedd4 interacts with Exon1 fragments of WT (WT Htt, exon1-25Q) and mHtt (mHtt, exon1-46Q) (Figure 1A), as well as with a longer mHtt fragment containing N-terminal 480 aa (Htt480-68Q) (Figure 1B). In the mouse brain, Nedd4 expression level was relatively low in the striatum and high in the cerebellum, as determined by immunoblot analysis (Figure 1, C and D) and immunofluorescence staining (Supplemental Figure 1; supplemental material available online with this article; https://doi.org/10.1172/jci.insight.181013DS1). Co-IP analysis of whole mouse brain lysates revealed an interaction between endogenous Nedd4 and endogenous Htt (Figure 1E). Since Nedd4 is an E3 Ub ligase, we examined the ubiquitination status of mHtt (Htt571-72Q) in the presence of empty vector, WT Nedd4, or ligase-dead Nedd4 (Nedd4 CS). These experiments revealed that ubiquitination of mHtt increased upon overexpression of Nedd4 as compared with empty vector or ligase-dead Nedd4 (Figure 1F), suggesting that Htt is ubiquitinated by Nedd4. As a negative control, we examined p53 and found that its ubiquitination was unaffected by Nedd4 ligase activity (Supplemental Figure 2A), whereas p53 is readily ubiquitinated by MDM2, a well-characterized E3 ligase for p53 (Supplemental Figure 2B). Given the presence of Ub^+^ high molecular weight (HMW) species of immunoprecipitated Htt (Figure 1F), we examined if Htt is polyubiquitinated or monoubiquitinated at multiple lysine sites by Nedd4 by using WT Ub or K0 Ub (K0 Ub) constructs. K0 Ub is a Ub variant in which all 7 lysine residues are mutated to arginine, thereby preventing the formation of Ub-to-Ub linkages and eliminating polyubiquitin chain branching. As a result, K0 Ub can support only monoubiquitination of substrate proteins. If a substrate protein is monoubiquitinated at multiple lysine residues, cotransfection with either WT Ub or K0 Ub will produce a similar pattern of HMW species, reflecting multiple single Ub additions. In contrast, if a substrate protein is polyubiquitinated at 1 or more lysine residues, cotransfection with WT Ub will generate characteristic smeared HMW bands due to the formation of branched polyubiquitin chains. However, cotransfection with K0 Ub will restrict modification to a single Ub per lysine, thereby eliminating the smeared HMW pattern. Thus, comparing the HMW profiles generated in the presence of WT versus K0 Ub provides a straightforward means to distinguish whether a protein of interest undergoes monoubiquitination or polyubiquitination. Using GFP as control substrate for polyubiquitination (Supplemental Figure 3), we found that overexpression of HA-tagged WT Ub led to generation of HMW species, whereas K0 Ub led to abrogation of these HMW bands. When the ubiquitination status of mHtt (Htt480-68Q) was examined with this assay, the overexpression of either WT Ub or K0 Ub resulted in similar pattern of HMW species, suggesting that Htt is monoubiquitinated at multiple lysine sites (Figure 1G).

**Figure 2:**
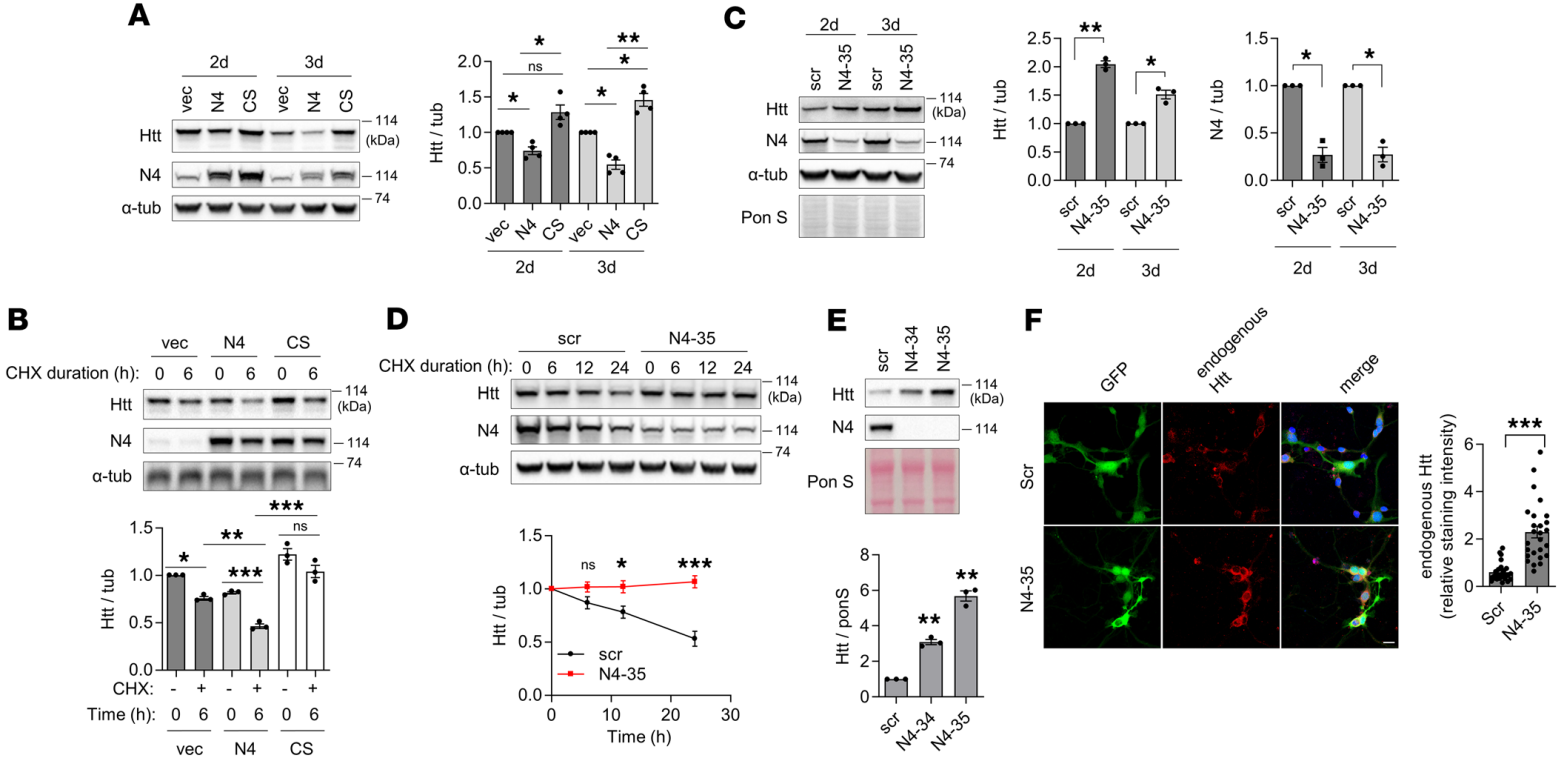
Nedd4 regulates Htt level and degradation in N2a and primary neuronal cells. (**A**) Htt levels are reduced by overexpression of Nedd4 in a ligase activity–dependent manner in N2a cells. Cells were transfected with Htt571-72Q together with vector (vec), Nedd4 (N4), or Nedd4 CS (CS), and analyzed by Western blot 2 and 3 days after transfection. *n* = 4, one-way ANOVA with Tukey’s multiple-comparison test. (**B**) Htt degradation is enhanced by overexpression of Nedd4 in a ligase activity–dependent manner in N2a cells. Cells were transfected with Htt571-72Q together with vector, Nedd4, or Nedd4 CS. Cycloheximide (CHX) 6-hour-chase experiment was followed by Western blot. *n* = 3, two-way ANOVA with Tukey’s multiple-comparison test. (**C**) Nedd4 knockdown increases Htt levels in N2a cells. Cells were cotransfected with Htt571-72Q and scrambled (scr) or shNedd4-35 (N4-35) plasmid, harvested at the indicated time points, and analyzed by Western blot. *n* = 3, one-sample *t* test. (**D**) Nedd4 knockdown impairs Htt degradation in N2a cells. Cells were transfected with scr or N4-35 plasmid. Forty-eight hours later, they were transfected with Htt571-72Q, subjected to 6-, 12-, and 24-hour CHX-chase experiment, harvested and analyzed by Western blot. *n* = 3, two-way ANOVA with Šídák’s multiple-comparison test. (**E**) Nedd4 knockdown increases Htt levels in mouse primary cortical neurons. Cells were transduced with lentivirus expressing Htt571-72Q together with scrambled (scr), shNedd4-34 (N4-34), or N4-35 lentivirus, harvested, and analyzed by Western blot. *n* = 3, one-way ANOVA with Dunnett’s multiple-comparison test. (**F**) Nedd4 knockdown increases endogenous Htt levels in mouse primary cortical neurons. Twenty-four hours after plating, cells were transduced with lentivirus expressing GFP and either scr or N4-35, cultured for an additional 6 days, and immunostained with anti-Htt antibody (5656S). Htt intensity was quantified in double-transduced cells (*n* = 26 fields, 2-tailed Student *t* test; scale bar: 20 μm). α-tubulin (α-tub), loading control. *P < 0.05; **P < 0.01; ***P < 0.001; NS, not significant.

Importantly, we found that Htt level was reduced upon Nedd4 overexpression in a ligase activity- dependent manner (Figure 1F and Figure 2A), suggesting that Htt may be degraded upon Nedd4-mediated ubiquitination. Moreover, expression of Nedd4 reduced the levels of both full-length Htt with normal and expanded polyQ repeats in HEK 293 cells stably expressing these proteins (27) (Supplemental Figure 4). The downregulation was specific for Htt, since another Nedd4 substrate, Mdm2, was upregulated by Nedd4 in an E3 ligase activity-dependent manner, as previously reported (28) (Supplemental Figure 5, A and B), whereas the levels of GFP were unaffected (Supplemental Figure 5, C and D). Using cycloheximide (CHX) chase to assess protein degradation in the absence of ongoing protein synthesis, we found that the degradation rate of Htt was enhanced by Nedd4 compared with empty vector or Nedd4 CS (Figure 2B). Furthermore, shRNA-mediated silencing of endogenous Nedd4 led to increased levels of Htt (Figure 2C), whereas silencing of endogenous Nedd4 abrogated Htt degradation in CHX chase experiment (Figure 2D). To determine if this regulation of Htt levels was also operational in neurons, lentiviruses carrying mHtt gene and scrambled or Nedd4 shRNA were transduced into primary neurons. These experiments revealed that mHtt levels were dramatically increased upon silencing of Nedd4 in neurons (Figure 2E), further demonstrating that Nedd4-mediated ubiquitination promotes degradation of mHtt. We also observed a significant increase in endogenous Htt levels following Nedd4 knockdown in primary mouse cortical neurons, as shown by immunofluorescence staining of endogenous Htt (Figure 2F), suggesting that Nedd4 regulates Htt abundance in neurons.

Ubiquitination of cellular proteins may lead to their degradation via proteasomal or autophagy/lysosomal pathways. Thus, we sought to determine the degradation pathway(s) involved in Htt clearance by inhibiting proteasome (using bortezomib) or lysosomes (using bafilomycin A1) during CHX chase experiments. The efficacy of bortezomib and bafilomycin A1 was verified by monitoring the accumulation of ubiquitinated proteins and of p62, respectively (Supplemental Figure 6, A and B).

Nedd4 overexpression led to faster degradation of mHtt compared with an empty vector or Nedd4 CS (Figure 2B). This enhanced degradation was partially reversed by bafilomycin A1 but largely unaffected by bortezomib (Supplemental Figure 6A), suggesting that Nedd4-mediated ubiquitination of Htt targets Htt preferentially to lysosomal degradation pathway.

### Nedd4 regulates mHtt aggregation and toxicity.

**Figure 3:**
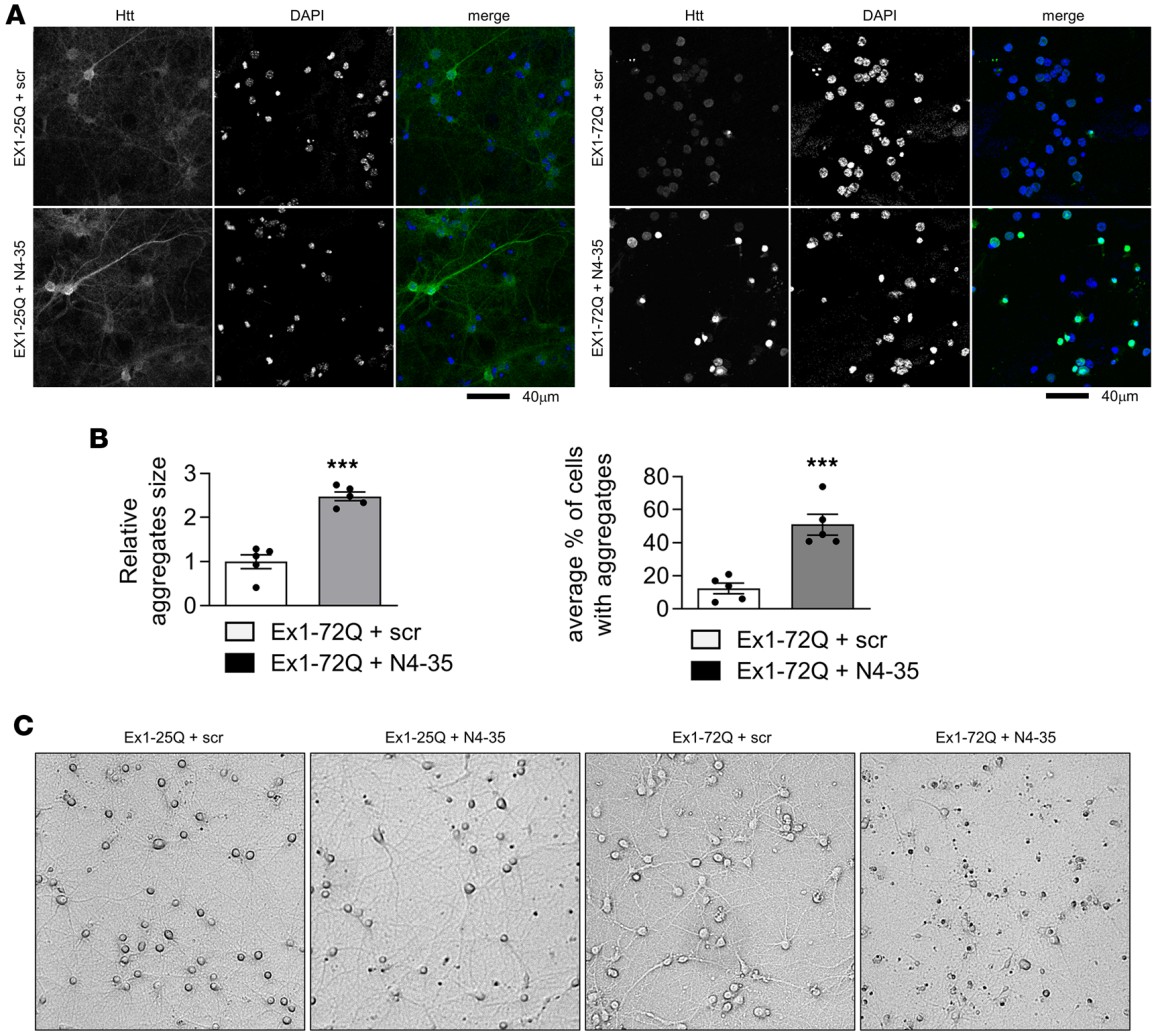
Nedd4 regulates mHtt aggregation and toxicity in primary cortical neurons. (**A**) Nedd4 KD increases mHtt aggregation in primary cortical neurons. Mouse primary cortical neurons were transduced with lentivirus expressing Htt Exon1-25Q or -72Q together with scrambled (scr) or shNedd4-35 (N4-35). Cells were fixed and immunostained for Htt followed by confocal microscopy. DAPI was included in mounting medium to stain nuclei. (**B**) Nedd4 KD increases the size and the number of mHtt aggregates in primary cortical neurons. *n* = 5 microscopic fields for each, 2-tailed unpaired *t* test (***P < 0.001). (**C**) Nedd4 KD enhances Htt toxicity in an mHtt expression-dependent manner in primary cortical neurons. Mouse primary cortical neurons were transduced with lentivirus expressing Htt Exon1-25Q or -72Q together with scrambled (scr) or shNedd4-35 (N4-35). Pictures were taken with bright-field microscope to show cell morphology.

To assess the effects of endogenous Nedd4 on mHtt aggregation, we coexpressed exon 1 Htt (25Q or 72Q) with scrambled or Nedd4 shRNA in primary cortical neurons and analyzed Htt aggregation by confocal microscopy (Figure 3A). These experiments revealed that both the size and the number of mHtt aggregates were significantly increased upon Nedd4 knockdown (Figure 3B). Furthermore, bright-field microscopy of neurons expressing Htt Ex1-25Q or -72Q with either scrambled or Nedd4 shRNA showed that Nedd4 KD enhanced mHtt toxicity, leading to abnormal neuronal morphology and signs of cellular distress (Figure 3C). Immunoblot analysis of lysates from lentivirus-infected neurons showed a near-complete loss of endogenous Nedd4 (Supplemental Figure 7A). Notably, the primary neuronal cultures consisted mainly of NeuN^+^ neurons with minimal GFAP staining, indicating that lentiviral gene expression was primarily confined to neurons (Supplemental Figure 7B). To investigate which aspects of neuronal pathology were exacerbated by Nedd4 reduction, we performed immunofluorescence staining to assess neuronal morphology in primary neurons. In neurons coinfected with mHtt and Nedd4 shRNA, we observed a significant decrease in neurite length, as indicated by MAP2 staining. These results support the conclusion that Nedd4 reduction exacerbates the neurotoxic effects of mHtt (Supplemental Figure 8).

**Figure 4:**
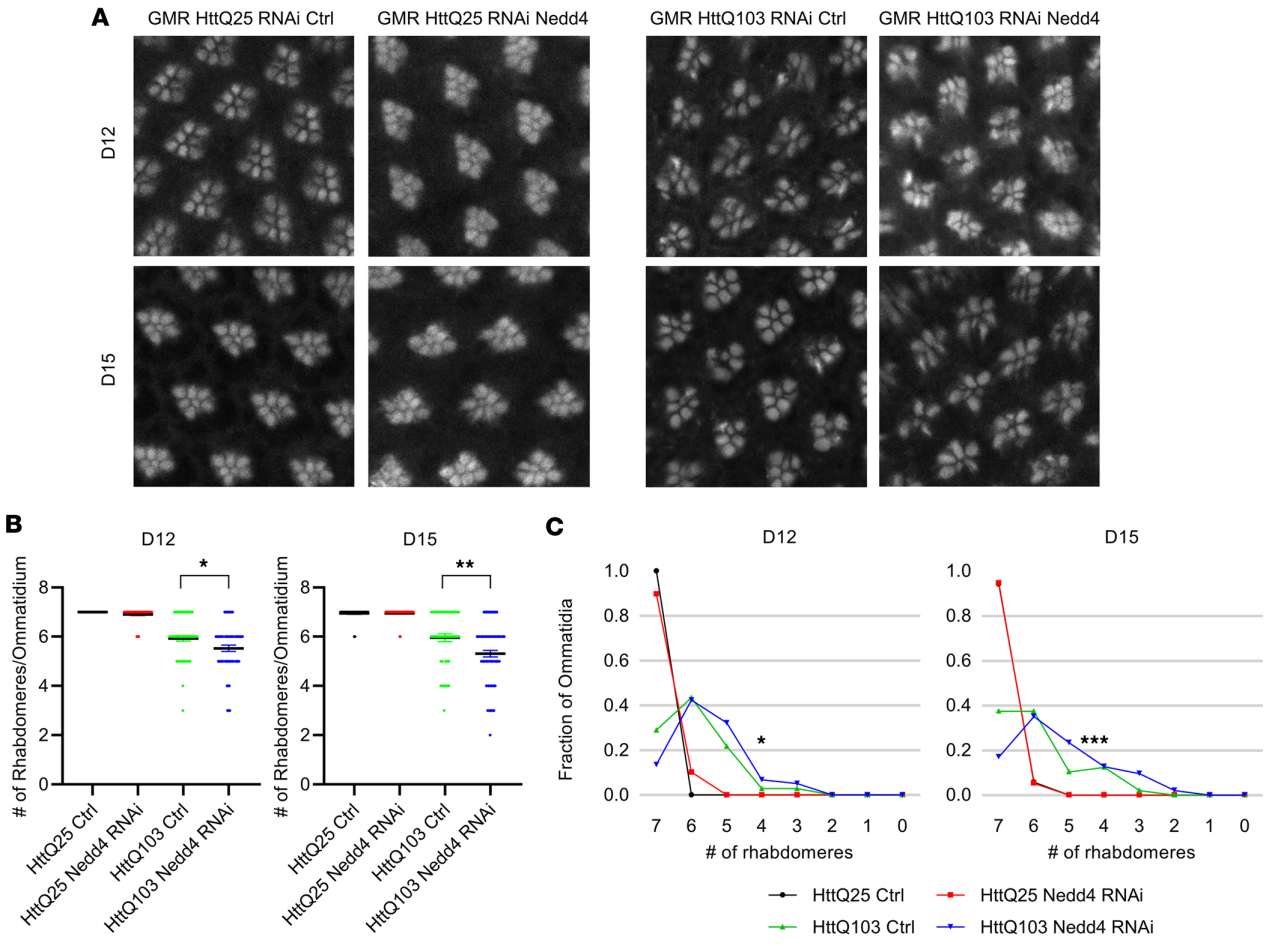
Knockdown of Nedd4 enhances the retina degeneration induced by Htt Exon1-Q103. (**A**) Representative images of the retina from GMR>HttQ25 or GMR>HttQ103 with control (Ctrl RNAi) or Nedd4 knockdown (Nedd4 RNAi) flies are shown. Male flies were collected within the first 24 hours after eclosion and aged on the regular fly medium. Retinas were dissected after aging the flies for 12 or 15 days (D12 or D15). Actin in rhabdomeres was stained by phalloidin to visualize the integrity of the ommatidia. (**B**) The number of rhabdomeres in each ommatidium was counted and the average number of ommatidium ± SEM for each genotype at both D12 and D15 is shown. *n* = 79, 49, 69, and 59 from left to right for D12. *n* = 52, 75, 48, and 94 from left to right for D15. Mann-Whitney *U* test (*P < 0.05, **P < 0.01). (**C**) Distribution of the fraction of ommatidia that contain a certain number of rhabdomeres was shown for each genotype at both D12 and D15. Mann-Whitney *U* test was performed to assess the significance in the difference of ommatidia integrity between genotypes. GMR>HttQ103 is significantly different from GMR>HttQ25 at both D12 and D15 (*P* < 0.005, not labeled on the plots). *P* values for Mann-Whitney *U* test between GMR>HttQ103 Ctrl RNAi and GMR>HttQ103 Nedd4 RNAi are indicated on the plots (*P < 0.05, ***P < 0.005).

To examine whether Nedd4 affects mHtt toxicity in vivo, we performed eye-specific silencing of Nedd4 using RNAi in *D*. *Melanogaster* with eye-specific expression of Htt. Retinal degeneration of Drosophila has been used to study different types of neurodegenerations (29–32). Several mHtt transgenes driven by a GAL4 expressed in the eye (GMR-GAL4) have been shown to induce retinal neurodegeneration, affecting the number and arrangement of rhabdomeres in the ommatidium (32–35). In our fly assays (Figure 4), human Ex1-25Q or 103Q was used. While GMR>HttQ25 flies displayed completely intact ommatidium structure (7 intact rhabdomeres per ommatidium), we observed disordered arrangement and decreased number of rhabdomeres (6 rhabdomeres) in the ommatidia of GMR>HttQ103 flies both at day 12 and at day 15, as expected. Interestingly, Nedd4 KD did not cause any impairment in GMR>HttQ25 flies. However, it significantly enhanced retinal degeneration in GMR>HttQ103 flies, reducing the number of intact rhabdomeres per ommatidium to 5.5. Silencing of Nedd4 was confirmed by qPCR (Supplemental Figure 9). Of note, Nedd4 RNAi decreased the level of Nedd4 RNA from the entire heads by 50% in flies with panneuronal knockdown of Nedd4 (where elav-GAL4 driver was used), indicating that the KD efficiency of Nedd4 in neurons alone was much higher than 50% since the total RNA extracted from the heads included RNAs from nonneuronal cells as well. Together, these data suggest that Nedd4 ubiquitinates mHtt, thus contributing to reduction in mHtt levels, its aggregation, and toxicity.

### Nedd4 level is reduced in mHtt-expressing primary neurons and brains of HD mouse model through a mechanism involving mTORC1.

**Figure 5:**
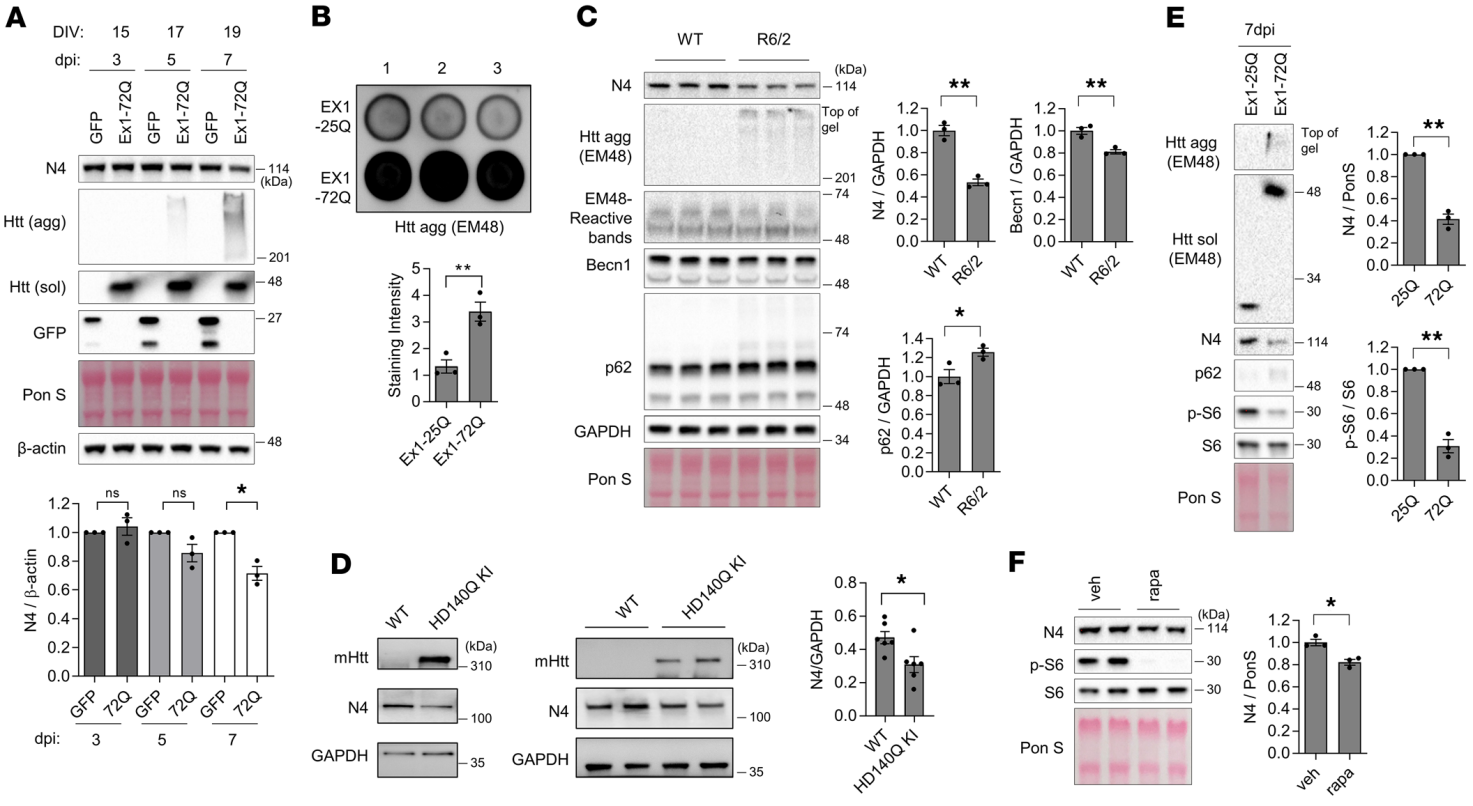
mHtt expression reduces Nedd4 levels in neurons and HD brains through impaired mTORC1 activity. (**A**) Overexpression of mHtt reduces Nedd4 levels in rat primary cortical neurons. Cells were transduced with lentivirus expressing GFP or Htt Exon1-72Q, harvested on indicated days, and analyzed by Western blot. GFP, aggregated (agg) and soluble (sol) Htt were detected. *n* = 3, one-sample *t* test. (**B**) Filter trap-dot blotting analysis of Htt aggregation. Twenty-four hours after plating, mouse cortical neurons were transduced with lentivirus expressing either Htt Ex1-25Q or Htt Ex1-72Q and cultured for 6 days. Htt aggregates were detected by anti-EM48 antibody (MAB5374). Intensity of aggregated Htt was quantified (*n* = 3, two-tailed Student’s *t* test, *P* = 0.0091) and presented as mean values ± SEM. (**C**) Nedd4 levels are reduced in brains of R6/2 model of HD. WT and R6/2 cerebra were homogenized and analyzed by Western blot using antibodies against EM48, Beclin 1 (Becn1), and p62. *n* = 3, two-tailed unpaired *t* test. (**D**) Nedd4 levels are reduced in brains of HD140Q KI mice. Cortical lysates of WT and HD140Q KI were analyzed by Western blot using antibodies against Nedd4 and Htt. Nedd4 values were normalized by Gapdh (*n* = 6, two-tailed Student’s *t* test, *P* = 0.023). (**E**) mHtt overexpression in primary cortical neurons reduces phospho-S6 (p-S6) and Nedd4 levels. *n* = 3, one-sample *t* test. (**F**) Acute inhibition of mTORC1 activity by rapamycin reduces Nedd4 level in rat primary cortical neurons. Cells were treated with vehicle (veh) or 20 nM rapamycin (rapa) for 24 hours on DIV9, harvested, and analyzed by Western blot using antibodies against Nedd4, p-S6, and S6. *n* = 3, two-tailed unpaired *t* test. β-actin (actin), Gapdh and Ponceau S (Pon S) were used as loading controls. *P < 0.05; **P < 0.01; NS, not significant.

While performing experiments in primary neurons, we observed an unexpected decrease in Nedd4 level in neurons expressing Htt Ex1-72Q as compared with control neurons expressing an empty vector, GFP, or Htt Ex1-25Q (Figure 5A and data not shown). Lentiviral expression of Htt Ex1-72Q in primary neurons resulted in the formation of high–molecular weight aggregates, as detected by immunoblot analysis of Htt. Consistently, a filter trap assay — a classical method for assessing the aggregation of misfolded proteins (36) — revealed a significantly higher amount of mHtt aggregates in neurons expressing Ex1-72Q compared with those expressing Ex1-25Q (Figure 5B). In addition, we found a significant reduction in Nedd4 levels in brain samples from R6/2 mice, expressing Htt Ex1-150-200Q, as compared with WT mice (Figure 5C). Interestingly, this was accompanied by decreased Beclin 1 and increased p62 in R6/2 mouse brains compared with WT controls (Figure 5B), suggesting an impairment of autophagy in this mouse model of HD. Similarly, immunoblot analysis of HD 140Q knock-in (KI) mice revealed a significant decrease in Nedd4 levels in the striatum compared with WT controls (Figure 5D). The HD 140Q-KI model expresses full-length mHtt at endogenous levels and is widely used in the HD research field (37–39).

We next attempted to elucidate the mechanism by which mHtt affects Nedd4 level. It has been shown previously that mTORC1 activity is reduced in HD, which contributes to autophagy impairment in HD (40). Moreover, mTORC1 was reported to regulate Nedd4 level in neurons as its downstream effector (41). We found that Htt Ex1-72Q–expressing neurons displayed reduced mTORC1 activity, as revealed by decreased phosphorylation of mTORC1 substrate S6, in comparison with Htt Ex1-25Q–expressing neurons (40) (Figure 5E). In addition, acute inhibition of mTORC1 by rapamycin led to reduction of Nedd4 levels in primary cortical neurons in agreement with the findings by Hsia et al. (41) (Figure 5F). Together, these results suggest that mHtt impairs mTORC1 activity, which in turn leads to reduced expression of Nedd4.

## Discussion

Our study revealed that the Ub ligase Nedd4 promotes clearance of mHtt and protects neurons from mHtt toxicity. We propose that mHtt and Nedd4 form a feedback loop in which mHtt downregulates Nedd4 through its inhibitory effect on mTORC1 activity, whereas reduced Nedd4 in turn leads to decreased ubiquitination and degradation of mHtt, ultimately leading to accumulation and toxicity of mHtt.

One of the toxic effects of mHtt is aberrant autophagic degradation, but the mechanism leading to defects in autophagy is incompletely understood. In this study, we showed that Nedd4 is downregulated in mHtt- expressing primary neurons and brains of mouse model of HD (R6/2 and HD 140Q KI) (Figure 5, A, C, and D). Since both mHtt expression and Nedd4 silencing result in aberrant autophagy (20, 42–44), Nedd4 might represent a molecular link between mHtt and autophagy impairment in HD. How does mHtt regulate Nedd4 levels? Kawabe and colleagues have reported that Nedd4 translation depends on mTORC1 activity (41). Moreover, a dramatic inhibition of mTORC1 activity has been observed in HD mouse and human brains (40). We found that mTORC1 activity was reduced in primary neuronal model of HD (Figure 5E), along with Nedd4 downregulation (Figure 5E). Hence, our data together with the above-mentioned studies suggest the existence of a cascade of events where mHtt triggers partial inactivation of mTORC1, which is followed by impaired Nedd4 translation and ultimately deficient autophagy. Current views on the role of mTORC1 suggest that its role in autophagy induction depends on the type of autophagy or cell type. While it has been established that mTORC1 inhibition is the major trigger for starvation-induced autophagy in dividing cells (45), there is also evidence that autophagy persists under mTORC1-stimulated conditions (46). Furthermore, mTORC1 stimulates autophagy in HD mouse brains and ameliorates the disease phenotypes, possibly through upregulation of Beclin-1 (40). It is therefore tempting to speculate that, while mTORC1 suppresses starvation-induced autophagy, it may at the same time promote selective autophagy, which is responsible for degradation of protein aggregates, including mHtt. Another not-mutually-exclusive possibility is that mTORC1 has different roles in neuronal versus nonneuronal cells. Interestingly, mounting evidence demonstrates that neuronal autophagic mechanisms differ from autophagy in other cells (47). For example, starvation and rapamycin-mediated inhibition of mTORC1 do not lead to robust induction of autophagy in neurons (48–50). This raises a possibility that selective autophagy is the dominant form of autophagy in neurons, whereas starvation-induced autophagy might not play a critical role in these cells. The reasons for this might be the necessity for neurons to continuously maintain proteostasis and clearance of misfolded proteins due to their postmitotic status. In addition, neurons may be protected from starvation by the surrounding glial cells, which would then circumvent the need for starvation-triggered rescue mechanisms.

Manipulations of mHtt PTMs aiming to enhance mHtt clearance have been suggested in a number of prior studies. For instance, Ub ligase Hrd1 ubiquitinates mHtt and reduces its levels, the formation of inclusion bodies, and toxicity (51), while Parkin leads to more general ubiquitination and downregulation of polyQ-containing proteins (52). Cochaperone CHIP also promotes ubiquitination and degradation of mHtt accompanied with a decrease in toxicity (53). PTMs also determine the degradation pathway for the proteins: while Ub ligase E3A leads to proteasomal degradation of mHtt (54), acetylation at K444 tags mHtt for autophagic clearance (5). It will be of interest to examine the interplay of these PTMs, including Nedd4-mediated ubiquitination, in regulating neuronal levels of mHtt.

Together, our data suggest a complex interplay between mHtt and Nedd4 in neurodegeneration underlying HD. While Nedd4 ubiquitinates mHtt and targets it to degradation, mHtt downregulates Nedd4 through mTORC1-dependent pathways, and this downregulation further leads to damaging downstream effects such as impairment of basal autophagy. The resulting decrease in Nedd4 in turn stabilizes mHtt, leading to further accumulation of the toxic protein and hence to the reinforcement of the positive feedback loop. We suggest that restoring Nedd4 function might lead to interruption of this vicious cycle, in which case Nedd4 would represent a potential target for pharmacological intervention of HD.

## Methods

### Sex as a biological variable.

Sex was not considered as a biological variable. Both male and female mice were used in this study.

### Antibodies.

The following antibodies were used for Western blotting: Nedd4 (07-049, EMD Millipore), Nedd4 (21698-1-AP, Proteintech), GFP (G1544, Sigma), GFP (632381, TaKaRa), GFP (GTX-113617, Genetex), T7-Tag (#6885, Cell Signaling Technology), huntingtin (MAB5490, MAB5492, MAB2166, MAB5374, MAB1574, MilliporeSigma), huntingtin (BML-PW0595A, Enzo), Huntingtin (ab109115, Abcam), HA- Tag (#3724, Cell Signaling Technology), HA-Tag (MMS-101P, Covance), HA-tag (66006-2-lg, Proteintech), α-tubulin (T5168, Sigma), p62 (18420-1-AP, Proteintech), p62 (P0067, Sigma), p62 (ab56416, Abcam), Ub (P4D1) (sc-8017, Santa Cruz), GAPDH (MAB374, MilliporeSigma), GAPDH (60004-1-lg, Proteintech), β-actin (ab6276, Abcam), Beclin-1 (#3738, Cell Signaling Technology), phospho-S6 (Ser235/236, #2211, Cell Signaling Technology), S6 (#2217, Cell Signaling Technology), Mdm2 (sc-965, Santa Cruz), Mdm2 (27883-1-AP, Proteintech), p53 (21891-1-AP, Proteintech), Vinculin (MAB3574, Millipore), and HRP-conjugated goat anti-rabbit and goat anti-mouse IgG (H+L) secondary antibodies (111-035-144 and 115-035-146, Jackson ImmunoResearch Laboratories). The following antibodies were used for IP: GFP (AFP5002, Qbiogene), GFP (G1544, Sigma), and huntingtin (MAB5490, MilliporeSigma). The following antibodies were used for immunocytochemistry: huntingtin (MAB5492, MilliporeSigma), huntingtin (5656S, Cell Signaling Technology), Nedd4 (21698-1-AP, Proteintech), NeuN (ABN90, Millipore), GFAP (sc-33673, Santa Cruz), MAP2 (ab11267, Abcam), Alexa Fluor 488 Phalloidin (A12379, Thermo Fisher), and Alexa Fluor 488 donkey anti-mouse IgG(H+L) secondary antibody (A-21202, Thermo Fisher).

### Reagents.

The following reagents were purchased as indicated: DMEM (11995065, Thermo Fisher), L-Glutamine (25030081, Thermo Fisher), Penicillin-Streptomycin (15140122, Thermo Fisher), Neurobasal media (21103049, Thermo Fisher), B-27 Supplement (17504044, Thermo Fisher), N-ethylmaleimde (23030, Thermo Fisher), PMSF (36978, Thermo Fisher), ZeptoMetrix RETROtek HIV-1 p24 Antigen ELISA (22-156-700, Fisher), FBS (100-106, Gemini Bio), CHX (14126, Cayman Chemical), bafilomycin A1 (11038, Cayman Chemical), bortezomib (#2204, Cell Signaling Technology), Ponseau S (P7170, Sigma), rapamycin (R8781, Sigma), cOmplete mini EDTA-free protease inhibitor cocktail (11836170001, Sigma), Protein A-Sepharose (P3391, Sigma), Protein G-Agarose (P4691, Sigma), Vectashield mounting medium with DAPI (H-1200, Vector Laboratories), Lenti-X concentrator (631232, TaKaRa), RNase-free DNase (79254, Qiagen), and Ub aldehyde (U-201-050, R&D Systems).

### Animals.

WT C57BL/6J mice were obtained from Guangdong Medical Laboratory Animal Center (Guangzhou, China). The HD140Q knock-in mice were described previously. All animals were housed under standard conditions (12:12 light-dark cycle) in the specific pathogen-free facility in the Division of Animal Resources at Jinan University, with all experimental protocols approved by the IACUC (application no. 106747, approval no. 20250630-04).

Genomic DNA was isolated from tail biopsies using a commercial extraction kit (CWBIO, CW2094S). PCR genotyping was performed using the following primer sets: WT-forward 5′-GCG GCT GAG GGG GTT GA-3′, HD-forward 5′-ACT GCT AAG TGG CGC CGC GTA G-3′, and common reverse 5′-GAG GCA GCA GCG GCT GTG CCT G-3′. Biological sex was not factored into the research design or data analysis for this study.

### cDNAs, shRNAs and lentiviruses.

HA-WT-Ub (#17608), HA-K0-Ub (#17603), scramble shRNA (#1864), GFP-p53 (#12091), and psPAX2 (#12260) were purchased from Addgene. pLP/VSV-G was obtained from Invitrogen. shNedd4-34 (TRCN0000092434) and shNedd4-35 (TRCN0000092435) were purchased from Open Biosystems. pEGFP was purchased from Clontech. Htt-Exon1-25Q-GFP and Htt-Exon1-46Q-GFP were provided by Aleksey Kazantsev (NIH). Htt-480-68Q and Htt-571-72Q were described previously (5). T7-tagged WT Nedd4 and catalytically inactive Nedd4 (C853S) were provided by Daniela Rotin (Hospital for Sick Children, Toronto, Canada). Human Mdm2 was provided by Karen Vousden (The Beatson Institute for Cancer Research). Nedd4 and Nedd4 CS inserts were amplified by PCR from pRc/CMV-Nedd4 and -Nedd4 CS (provided by Daniela Rotin) and were subcloned into pcDNA3 at EcoRI/NotI sites, which were used in some experiments. EGFP insert was amplified by PCR from pEGFP-C1 (Clontech) and was subcloned into the lentiviral expression vector pER4 at NheI/NotI sites. PRK-NEDD4, PRK-MDM2, and PRK-GFP vectors were custom made by IGEbio. The following lentiviral vectors were custom-made and packaged by IGEbio. pLKO.1-EF1α-HTTex1-72Q-P2A-mCherry, pLKO.1-EF1α-HTTEx1-25Q–P2A-mCherry, and pLKO.1-U6-shRNA-NEDD4-EF1α-copGFP (targeting sequence: 5′-GCG CAA ACA TTC TGG AGG ATT-3′).

### Cell culture, transfection, and primary neuronal culture.

HEK-293FT (Invitrogen R70007) and murine neuroblastoma cell line N2A (ATCC CCL-131) were cultured in DMEM with 10% FBS. For the experiments using HEK293 cell lines stably expressing full-length Htt with either 23 or 120 glutamine repeats (23Q/120Q), HEK293 cell lines were cultured in medium containing 500 μg/mL hygromycin B (Invivogen, ant-hg-5). Primary cortical neurons were isolated either from E16 embryos of C57BL/6 mice or from E18 embryos of Sprague Dawley rats as described previously (55). Neurons were plated in 6-well plates (400,000 cells per well), 12-well plates (160,000 cells per well), or 24-well plates with 12 mm coverslips in them (80,000 cells per well). Neurons were cultured in Neurobasal media with glutamine, penicillin/streptomycin, and 2% B-27 supplement.

### Generation of lentiviruses and transductions.

Lentiviruses expressing Htt-Exon1-25Q, Htt-Exon1-72Q, GFP, scramble shRNA, sh Nedd4-34, and shNedd4-35 were generated in 293FT cells (Thermo Fisher) by cotransfecting lentiviral vectors with helper plasmids (psPAX2 and pLP3) using X-tremeGene HP DNA Transfection Reagent (MilliporeSigma).

Media were replaced with fresh media 12 hours after transfection. Virus-containing culture supernatants were collected 48 hours after transfection and concentrated using Lenti-X concentrator. Virus titers were determined using the RETROtek HIV-1 p24 Antigen ELISA kit (ZeptoMetrix). In total, 1 ng p24 was considered equivalent to 5,000 infectious particles. Primary neurons were transduced with lentiviruses at the multiplicity of infection (moi) of 4–5 for expression vectors and 1.5 for knockdown, respectively, on DIV 9–13. Transduced neurons were harvested or fixed at 3–8 dpi (days post-infections) as indicated in the figures unless otherwise indicated.

### Transfections.

All cells were transfected with Lipofectamine 2000 for cDNA expression and shRNA knockdown according to the manufacturer’s instructions. Media were completely replaced with fresh media 4 h after transfection unless otherwise indicated.

### Preparation of cell lysates and tissue homogenates.

Total cell lysates were prepared by lysing cells in 2X LaemmLi sample buffer containing 2-mercaptoethanol, then boiling for 5 min. For time-course experiments, cells were harvested at the indicated time by removing media and freezing the sealed tissue culture plates in –20°C. The samples were lysed at the same time after harvesting all the time-course samples. For tissue homogenate preparation, WT and R6/2 cerebrum samples (110 day-old female brains, frozen) were homogenized in RIPA buffer (50 mM Tris, pH 7.4, 150 mM NaCl, 1 mM EDTA, 1% Triton X-100, 0.5% sodium deoxycholate, 0.1% SDS, 1X protease inhibitor cocktail, 1 mM PMSF) using a tissuemiser homogenizer (Thermo Fisher) and then sonicated using a Q700 sonicator (Qsonica). Tissue homogenates were cleared by centrifugation at 21,000*g* for 15 minutes at 4°C. Protein concentrations were determined by BCA protein assay. In total, 30 μg of proteins were resolved by SDS-PAGE and analyzed by Western blotting. For the detection of Nedd4 in HD140Q KI mice, cortical tissues from HD140Q KI mice (16-month-old) were homogenized using a Dounce Tissue Grinder (Thermo Fisher, K8853000002) with 20–30 strokes. Tissue lysates were prepared in RIPA buffer (50 mM Tris, pH 8.0, 150 mM NaCl, 1 mM EDTA, 1 mM EGTA, 0.1% SDS, 0.5% sodium deoxycholate, 1% Triton X-100) supplemented with protease inhibitors (Mei5bio, MF182-plus-10), followed by 6 cycles of sonication. Proteins were separated on SurePAGE Bis-Tris gels (GenScript, M00652) and transferred to nitrocellulose membranes. After blocking with 5% nonfat milk for 1 hour at room temperature, membranes were probed with primary antibodies diluted in 3% BSA overnight at 4°C. The following day, membranes were washed 3 times with PBS and incubated with HRP-conjugated secondary antibodies in 5% nonfat milk for 1 hour at room temperature. Following additional PBS washes, protein signals were detected using ECL substrate (Millipore, WBKLS0500) and visualized with a Clinx ChemiScope 6300 imaging system.

### Co-IP assay.

For co-IP assay of Htt-Exon1-GFP and Nedd4, 293FT cells grown on 6-well plates were transfected with plasmids. Forty-eight hours after transfection, cells were washed in PBS, and lysed on ice for 30 minutes in co-IP lysis buffer (40 mM HEPES, pH 7.4, 120 mM NaCl, 1 mM EDTA, 10 mM pyrophosphate, 10 mM glycerophosphate, 50 mM NaF, 0.3% CHAPS, 1 mM PMSF, 1X EDTA-free protease inhibitor cocktail). Cell lysates were cleared by centrifugation at 21,000*g* for 15 minutes. Protein concentration was determined by BCA assay. Equal amounts of proteins were precleared with 80 μL of protein G beads (20% slurry) for 2 hours to remove proteins that associate with protein G beads nonspecifically.

IP was performed with 1 μg of AFP antibody, 80 μL of protein G beads (20% slurry) and precleared lysates at 4°C overnight with rotation. IP samples were washed 3 times with co-IP lysis buffer. Inputs and IP samples were resolved by SDS-PAGE and analyzed by Western blotting. For co-IP assay of Ntt480-68Q and Nedd4, N2a cells grown on 6 cm dishes were transfected with plasmids. Cells were lysed on ice for 30 minutes in co-IP lysis buffer 26 hours after transfection. IP followed by Western blot analysis was performed. For the detection of interaction between Htt and Nedd4 in mouse brain lysates (Figure 1C), cortical tissue from 5-month-old WT C57BL/6J mice and cells were lysed in the buffer (40 mM HEPES, pH 7.4, 120 mM NaCl, 1 mM EDTA, 10 mM glycerophosphate, 50 mM NaF, 1 mM PMSF and protease inhibitors). The lysates (500 μg) were precleared with Protein G beads (30 μL, Thermo Fisher 10004D) and were then incubated with primary antibodies overnight at 4°C. Antibody complexes were captured with Protein G beads (50 μL, 1 hour at 4°C), washed 3× with 1% lysis buffer, magnetically isolated, and eluted in SDS loading buffer (95°C, 10 minutes).

### Ub assay.

For assessment of Htt571-72Q ubiquitination by Nedd4, N2a cells grown on 6 cm dishes were transfected with 0.5 μg HA-Ub (HA-Ub), 2 μg Htt571-72Q, and 1.5 μg vector (pRc/CMV), Nedd4, or Nedd4 CS. Cells were washed in PBS and collected 50 hours after transfection. Cells were lysed in 390 μL of lysis buffer (25 mM Tris, pH 7.5, 137 mM NaCl, 5 mM KCl, 1.5 mM Na_2_HPO_4_, 1 mM CaCl_2_·2H_2_O, 0.5 mM MgCl_2_·6H_2_O) by pipetting up and down. In total, 10 μL of 20% SDS was then added to the lysates and boiled at 70°C for 10 minutes to denature proteins. After lysates were cooled on ice, 1.6 mL of Ub dilution buffer (50 mM HEPES, pH 7.4, 150 mM NaCl, 10% glycerol, 1% Triton X-100, protease inhibitor cocktail [Roche], 2mM N-ethylmaleimide, 1mM PMSF) was added to the lysates. In total, 10 μL of RNase-free DNase was added and lysates were mixed by flipping the tubes. Cell lysates were incubated at room temperature until the viscosity went away. Lysates were cleared by centrifugation at 21,000*g* for 20 minutes, and protein concentration was determined by BCA assay. Cleared lysates were precleared with 80 μL of protein G beads (20% slurry) for 2 hours with rotation at 4°C. IP was performed by incubating precleared lysates, 80 μL of protein G beads (20% slurry), and 1.2 μL of anti-Htt antibody (MAB5490) at 4°C overnight. IP samples were washed 3 times with 1mL RIPA buffer (50mM Tris, pH7.4, 150mM NaCl, 1mM EDTA, 1% Triton X-100, 0.5% sodium deoxycholate, 0.1% SDS). Inputs and IP samples were resolved by SDS-PAGE and analyzed by Western blotting. For determination of Htt480-68Q monoubiquitination, N2a cells grown on 6-well plates were transfected with 1.5 μg of GFP-Htt480-68Q and 0.5 μg of either WT HA-Ub or HA-K0-Ub. Twenty-five hours after transfection, cells were washed in PBS, lysed in 100 μL of lysis buffer (50 mM Tris, pH 7.4, 150 mM NaCl, 1 mM EDTA, 0.5% Triton X-100, 1% SDS, 500 nM TSA, 5 mM nicotinamide, 1X protease inhibitor cocktail, 1 mM PMSF, 118 nM Ub aldehyde, 5 mM NEM, 1 mM DTT), boiled for 5 minutes, and cooled on ice. In total, 1 mL of Triton X-100 buffer (50 mM Tris, pH 7.4, 150 mM NaCl, 1% Triton X-100, 5 mM NaF, 1 mM EDTA) was added to the lysates and incubated on ice for 30 minutes. Lysates were cleared by centrifugation at 21,000*g* for 15 minutes. Cleared lysates were precleared with 80 μL of protein G beads (20% slurry). IP was performed by rotating precleared lysates, 1 μg of anti- AFP antibody (AFP5002, Q-biogene) and 80 μL of protein G beads (20% slurry) at 4°C overnight. IP samples were washed 4 times with 1 mL RIPA buffer (50 mM Tris, pH 7.4, 150 mM NaCl, 1 mM EDTA, 1% Triton X-100, 0.5% sodium deoxycholate, 0.1% SDS). Inputs and IP samples were resolved by SDS- PAGE and analyzed by Western blotting. For determination of GFP polyubiquitination, N2a cells grown on 6-well plates were transfected with 3 μg of GFP and 1 μg of HA-Ub or HA-K0-Ub. Cells were harvested 28 hours after transfection and immunoprecipitated as for the Htt480-68Q ubiquitination assay. For assessment of p53 ubiquitination by Nedd4, N2a cells grown on 6 cm dishes were transfected with p53-GFP, HA-Ub, and either Nedd4 or Nedd4 CS. Cells were harvested 28 hours after transfection, and immunoprecipitated as for the Htt480-68Q ubiquitination assay except the antibody used for IP was anti-GFP antibody from Sigma (G1544), and protein A beads were used in place of protein G beads.

### CHX chase assay.

For CHX chase of Htt with overexpression of Nedd4 or Nedd4 CS, N2a cells grown on 6 cm dishes were transfected with 4 μg of Htt571-72Q and 4 μg of vector (pcDNA 3.1+), Nedd4 or Nedd4 CS. Ten hours after transfection, cells in 6 cm dishes were trypsinized, collected, resuspended, and replated in 12-well plates. Twenty-four hours after transfection, t=0 h samples were harvested. At the same time, CHX was treated to t=6 h samples. Thirty hours after transfection, t=6 h samples were harvested. For CHX chase of Htt by knockdown of Nedd4, N2a cells grown on 6cm dishes were transfected with 4 μg of scramble shRNA or sh Nedd4-35. Twenty-four hours after transfection, cells were trypsinized and replated in 12-well plates. Forty-eight hours after transfection, shRNA-transfected cells were transfected with 0.8 μg of Htt571-72Q. Twenty-four hours after Htt transfection, t=0 h samples were harvested, and CHX was treated to the rest of the samples.

Samples were harvested 6, 12, and 24 hours after CHX treatment. For CHX chase with or without proteasome or lysosome inhibition, N2a cells grown on 6 cm dishes were transfected with 4 mg of Htt571-72Q and 4 μg of vector (pcDNA3.1+), Nedd4, or Nedd4 CS. Ten hours after transfection, cells in 6 cm dishes were trypsinized, collected, resuspended, and replated in 12-well plates. Twenty-four hours after transfection, t=0 h samples were harvested. At the same time, CHX was treated to t=6 h samples together with either vehicle (DMSO), bortezomib, or bafilomycin A1. Thirty hours after transfection, t=6 h samples were harvested. Samples were lysed, boiled for 5 minutes, resolved by SDS-PAGE, and analyzed by Western blotting.

### Immunofluorescence staining of primary neurons.

Primary neurons were fixed with 4% formaldehyde in 1X PBS for 15 minutes at room temperature. Coverslips were washed 2 times with PBS, blocked, and permeabilzed at the same time by incubating in 10% normal goat serum, 0.1% BSA, and 0.1% Triton X-100 in 1X PBS for 1 hour, incubated in anti-Htt antibody (MAB5492, 1:500) in 3% BSA in PBS at 4°C overnight. Coverslips were washed 3 times with PBS and incubated in secondary antibody (donkey anti-mouse IgG-Alexa Fluor 488, 1:600, Invitrogen, catalog A21202) for 1 hour at room temperature in the dark. Coverslips were washed 3 times with PBS, mounted using Vectashield mounting medium with DAPI, imaged using Leica DMI4000B confocal microscope and analyzed using ImageJ software (NIH).

### Immunofluorescence staining of mouse brain section.

Five-month-old WT C57BL/6J mice were anesthetized with isoflurane and transcardially perfused with warm saline (0.9%) followed by ice-cold 4% PFA in 0.1M PB. Brains were postfixed in 4% PFA overnight, then in 30% sucrose for 48 hours. After embedding in OCT compound (Sakura, 4583), 30 μm sections were obtained using a cryostat (Thermo Fisher). Sections and primary neurons were permeabilized with 0.5% Triton X-100/PBS (30 minutes) and blocked for 1 hour at room temperature. Primary antibody incubation was performed overnight at 4°C. After PBS washes, samples were stained with fluorescent secondary antibodies (1 hour) and DAPI (10 minutes), then imaged using a Zeiss AX10 or Olympus FV3000 microscope.

### Ommatidium staining and imaging.

Phalloidin staining for ommatidium was performed as described previously (34). Briefly, flies expressing Nedd4 RNAi with GMR-GAL4 (Nedd4 TRiP) or GMR-GAL4 only in the genetic background control for Nedd4 RNAi (TRiP Ctrl) were raised under 12:12 light/dark cycle, 25°C. Flies were collected within 1 day after eclosion and aged for 12 or 15 days prior to dissection. Adult eyes were dissected in PBS (137 mM NaCl, 2.7 mM KCl, 10 mM Na_2_HPO_4_ and 1.8 mM KH_2_PO_4_); then, retinas were fixed in 4% formaldehyde solution for 1 hour. Retinas were washed 5 times with PBS containing 0.3% Triton X-100 and were then kept in 400 μM phalloidin in PBS containing 0.3% Triton X-100 and 5% normal goat serum at 4°C overnight on a shaker. Retinas were washed 5 times with PBS containing 0.5% Triton X-100 and mounted in 80% glycerol. Images were taken with Nikon C2 confocal microscope. The number of rhabdomeres in each ommatidium was quantified in a blinded manner.

### Fly stock.

GMR-GAL4, Nedd4 RNAi (BL34741, y[1] sc[*] v[1] sev[21]; P{y[+t7.7] v[+t1.8]=TRiP.HMS01221}attP2) and its genetic control (BL36303, y[1] v[1]; P{y[+t7.7]=CaryP}attP2) were obtained from Bloomington Stock Center. UAS-HttQ25 and UAS-HttQ103 with C-terminal GFP fusion were provided by Norbert Perrimon (Harvard Medical School, Cambridge, MA, USA).

### qPCR.

Flies expressing Nedd4 RNAi with elav-GAL4 (Nedd4 TRiP) or elav-GAL4 only in the genetic background control for Nedd4 RNAi (TRiP Ctrl) were raised under 12:12 light/dark cycle, 25°C. Flies were collected within 5 days after eclosion. Heads from ~45 flies were separated for RNA purification. RNA was extracted using Trizol reagent by according to the manufacturer’s protocol and treated with DNase (Promega) and cDNA synthesized using SuperScript III cDNA system. qPCR was done using Bio-Rad Sybr Green. CT values were normalized to Rp49 as the internal control for calculation of relative RNA abundance.

### Statistics.

All values in figures and text refer to mean ± SEM unless otherwise stated. Statistics and graphing were performed using GraphPad Prism software. Data were analyzed using unpaired 2-tailed Student *t* test (for 2 datasets) or 1-way ANOVA with Tukey’s post hoc test (for multiple data sets) unless otherwise indicated. The difference between the numbers of rhabdomere in each ommatidium was tested with Mann-Whitney *U* test. A *P* value less than 0.05 was considered significant.

### Study approval.

All animal experiments were conducted in accordance with institutional guidelines and were approved by the Institutional Animal Care and Use Committee (IACUC) of Jinan University (application no. 106747; approval no. 20250630-04).

### Data availability.

Values for all data points found in graphs are in the Supporting Data Values file. All data needed to evaluate the conclusions in the paper are present in the paper and/or the supplemental materials.

## Author contributions

Conceptualization was contributed by HJ, SY, and DK. Methodology, analysis, and investigation were contributed by HJ, YQ, FX, KT, MJK, NM, and KH. Supervision was contributed by HJ, RA, SY, and DK. Review and editing were contributed by HJ, YQ, FX, KT, MJK, RA, SY, and DK. Funding acquisition was contributed by FX, RA, SY, and DK.

## Funding support

This work is the result of NIH funding, in whole or in part, and is subject to the NIH Public Access Policy. Through acceptance of this federal funding, the NIH has been given a right to make the work publicly available in PubMed Central.

- NIH NS122257 (to DK)
- DARPA (D12AP00023) and NIH(1R21 NS110420) (to RA)
- Training Grant in Circadian and Sleep Research (NIH T32HL007909; to FX)
- National Science Foundation of China (82471437; to SY)
- K. C. Wong Education Foundation support (to SY)

## Supplementary Material

Supplemental data

Unedited blot and gel images

Supporting data values

## Version 1 02/23/2026

Electronic publication
