# Supplementary material for: Ubiquitin ligase Nedd4 regulates the abundance and toxicity of mutant huntingtin
Source: JCI Insight. 2026 Feb 23;11(4):e181013. doi: 10.1172/jci.insight.181013 (PMC12956003; doi:10.1172/jci.insight.181013)
Supplement: Supplemental data [file jciinsight-11-181013-s045.pdf]

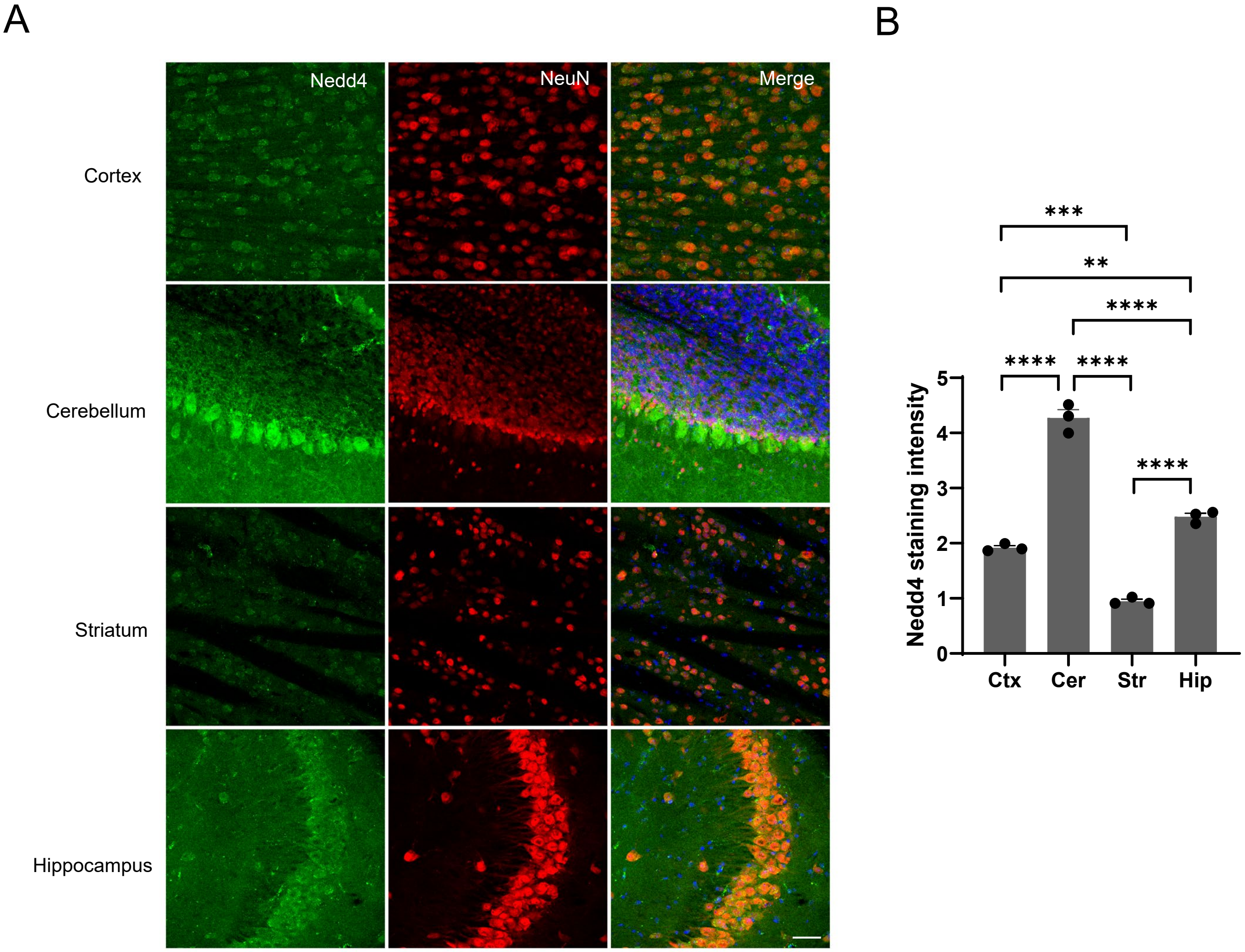

Supplemental Figure 1. Nedd4 expression in different brain regions of mice.

(**A**) Double immunostaining of Nedd4 and NeuN in different brain regions of mice (scale bar: 100 μm).

(**B**) Quantitative results of Nedd4 staining intensity in different brain regions of mice ($n = 3$, one-way ANOVA with Tukey post-tests, Ctx vs Cer, $p < 0.0001$; Ctx vs Str, $p = 0.0002$; Ctx vs Hip, $p = 0.0072$; Cer vs Str, $p < 0.0001$; Cer vs Hip, $p < 0.0001$; Str vs Hip, $p < 0.0001$). *, $p < 0.05$; **, $p < 0.01$; ***, $p < 0.001$; ****, $p < 0.0001$. Data are presented as mean values ± SEM.

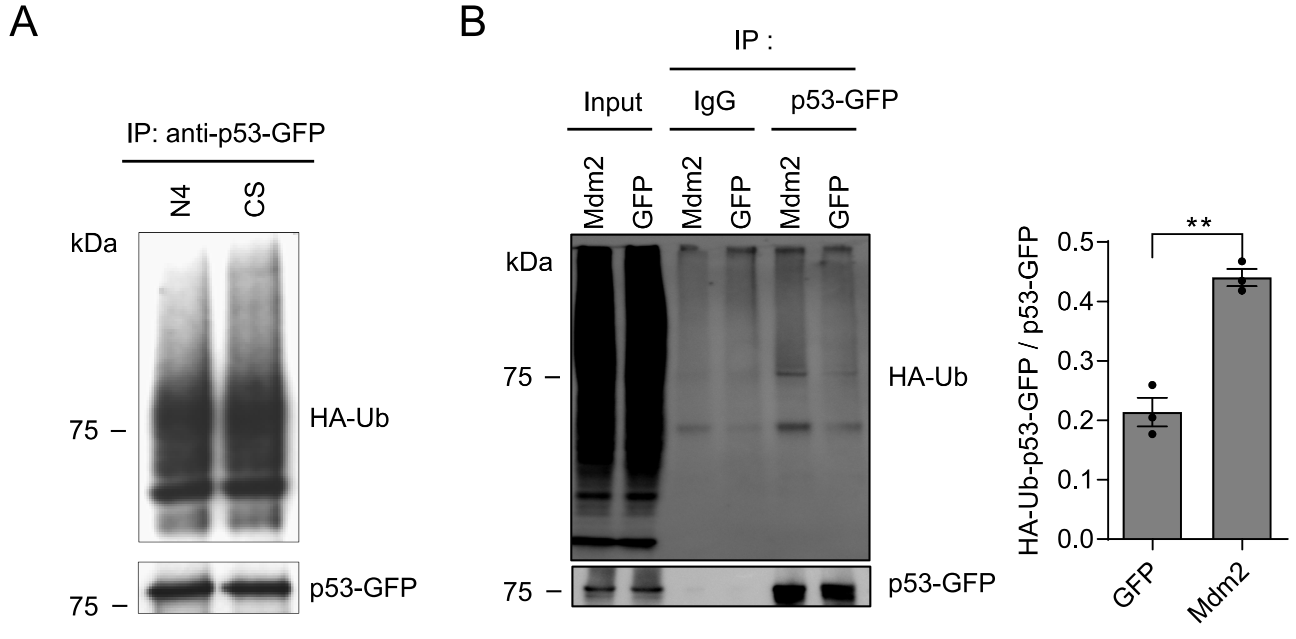

Supplemental Figure 2. p53 ubiquitination is unaffected by Nedd4 ligase activity.

(**A**) N2a cells were transfected with p53-GFP, HA-ubiquitin, together with wild type Nedd4 (N4) or inactive Nedd4 mutant (CS). Samples were collected 28 h post transfection, and immunoprecipitation (IP) performed under denaturing condition followed by western blot analysis using indicated antibodies. HA-Ub, N4 and CS stand for HA-ubiquitin, Nedd4 and Nedd4 CS, respectively.

(**B**) N2a cells were co-transfected with HA-Ub and p53-GFP constructs, along with either MDM2 or GFP plasmids by using Lipofectamine 3000 reagent (Invitrogen). At 24 h post-transfection, cells were treated with the proteasome inhibitor MG132 (10 µM; Solarbio, IM0310) for 12 h prior to collection for ubiquitination analysis. p53 antibody was used to pull down p53-GFP. IgG was used for control. p53-GFP and HA-ubiquitinated p53-GFP were analyzed in the pulldown lysate. Quantitative analysis of ubiquitinated p53-GFP normalized by p53-GFP pulled down (n = 3, two-tailed student t test, p = 0.0013).

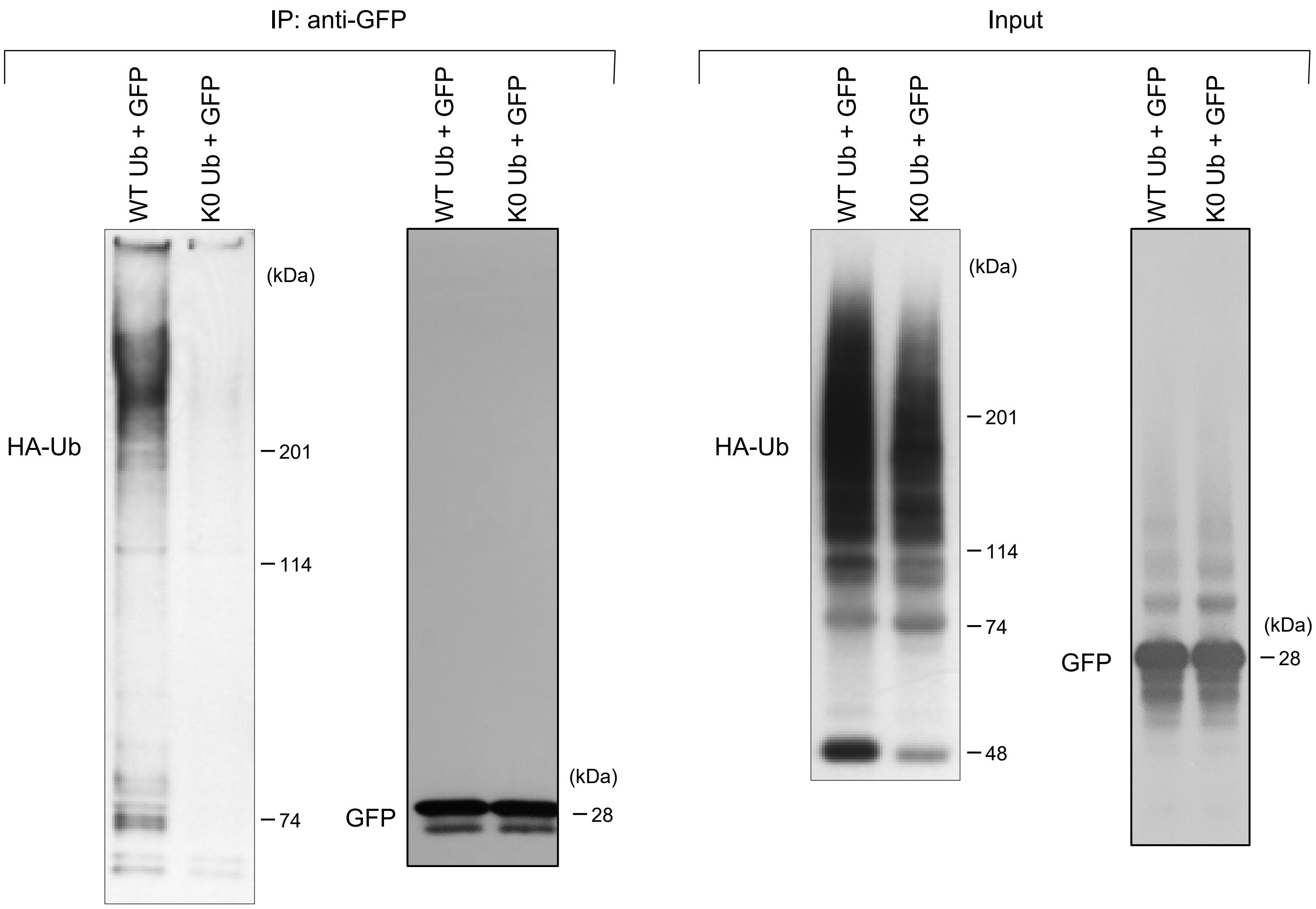

Supplemental Figure 3. Polyubiquitination of GFP is demonstrated by the presence of high molecular weight (HMW) species of ubiquitinated GFP only with overexpression of wild-type ubiquitin, but not with K0 ubiquitin.

N2a cells were transfected with GFP together with wild-type ubiquitin (WT Ub) or K0 ubiquitin (K0 Ub). Samples were collected 28 h post transfection, and IP performed under denaturing condition followed by western blot analysis.

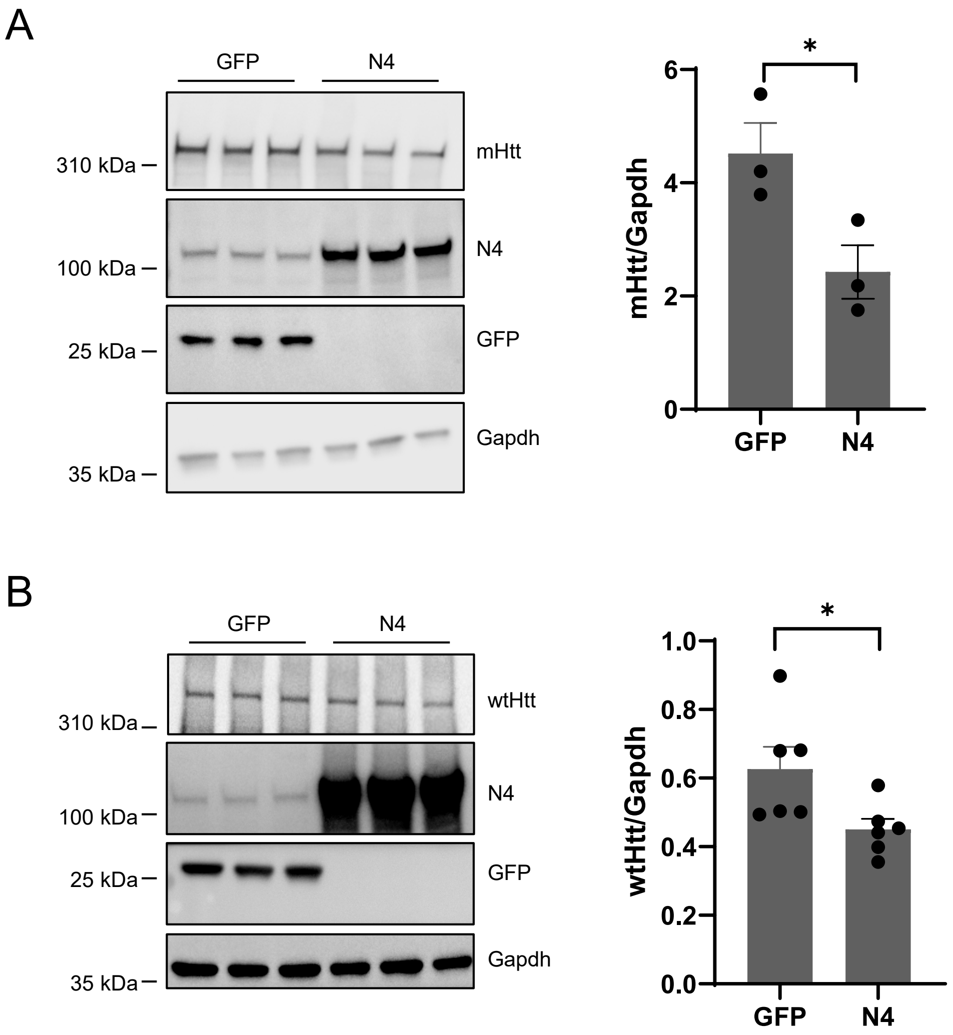

Supplemental Figure 4. Effects of Nedd4 on mHTT and wtHtt in HEK293 cells.
HEK293 cells stably expressing HTT with either 23Q or 120Q were transfected with NEDD4 or GFP plasmids by using Lipofectamine 3000 reagent (Invitrogen). Cells were collected 48 hours post-transfection for subsequent analysis.

(**A**) HEK293 cells stably expressing full-length mHtt were transfected with Nedd4 (N4) or GFP plasmids. Western blotting analysis of mHtt expression. Quantitative analysis of mHtt normalized by Gapdh ($n = 3$, two-tailed student t test, $p = 0.0431$).

(**B**) HEK293 cells stably expressing full-length wtHtt were transfected with Nedd4 (N4) or GFP plasmids. Western blotting analysis of wtHtt expression. Quantitative analysis of wtHTT normalized by Gapdh ($n = 6$, two-tailed student t test, $p = 0.0351$). *, $p < 0.05$. Data are presented as mean values $\pm$ SEM.

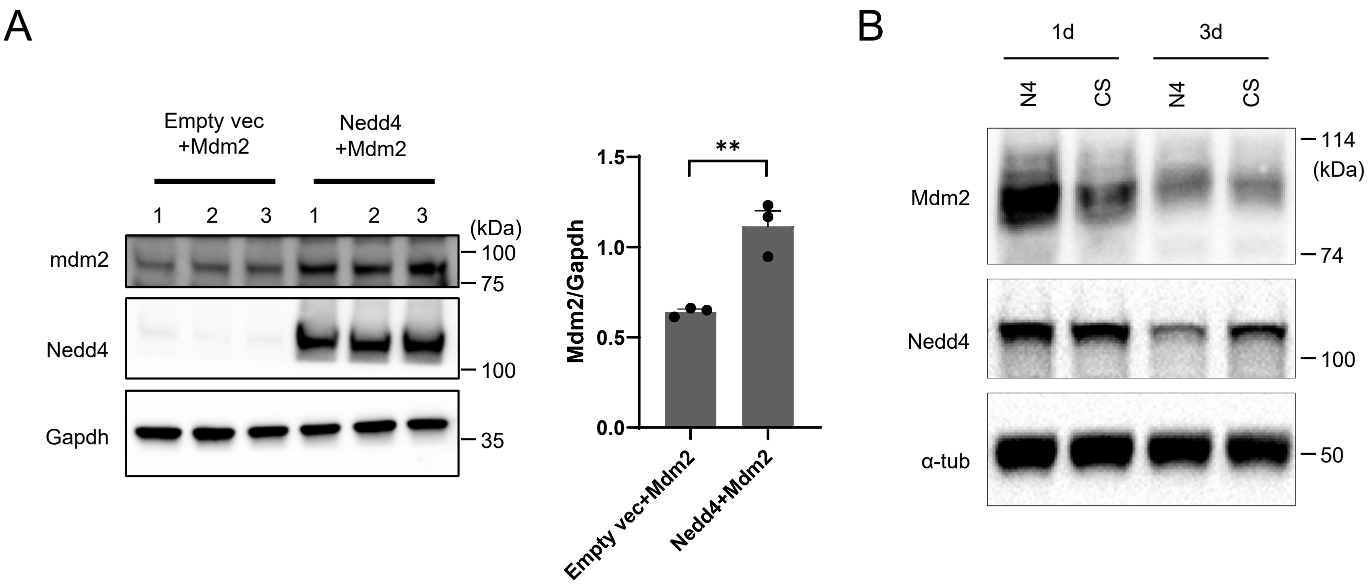

Supplemental Figure 5A and 5B. Mdm2 level is increased by Nedd4 ligase activity.

(**A**) N2a cells were transfected with Mdm2/Nedd4, or Mdm2/empty vector by using Lipofectamine 3000 reagent (Invitrogen). Cells were then harvested 48 hours post- transfection. Western blotting analysis of Mdm2 and Nedd4. Gapdh was used as a loading control. Quantitative analysis of Mdm2 normalized by Gapdh. (n = 3, two-tailed student t test, **, $p < 0.01$; ****. Data are presented as mean values ± SEM.

(**B**). Mdm2 level is increased by Nedd4 ligase activity. N2a cells were transfected with Mdm2 together with wild type Nedd4 (N4) or inactive Nedd4 CS (CS). Samples were collected at indicated time and western blot analysis performed. α-tubulin was used as loading control.

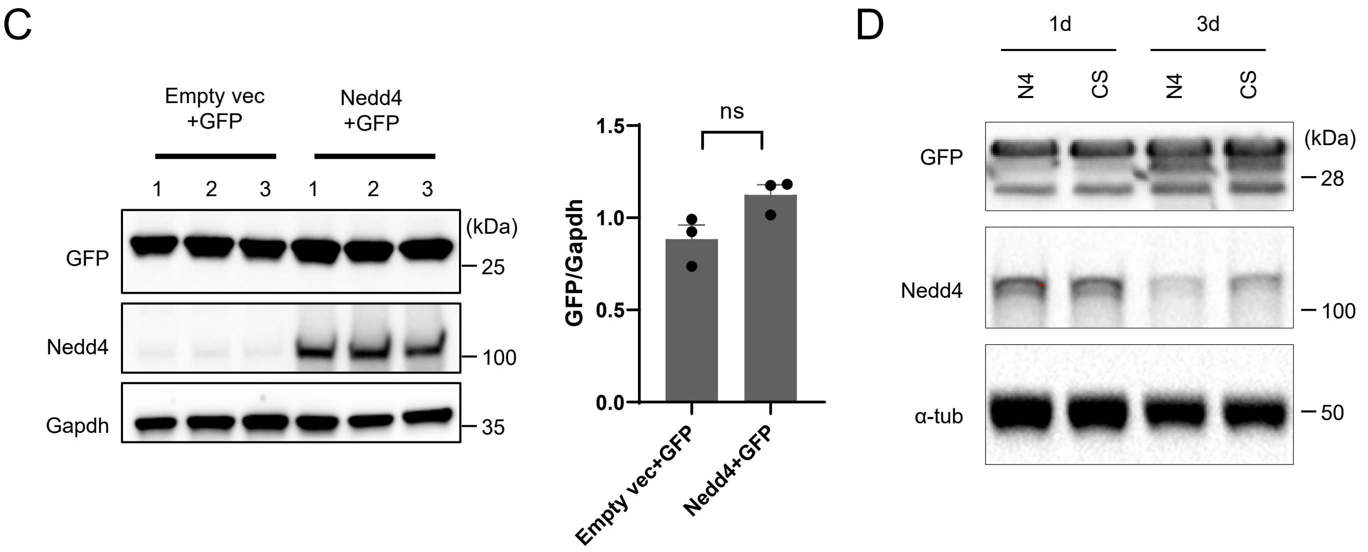

Supplemental Figure 5C and 5D. **GFP level is unaffected by Nedd4 ligase activity.**

(**C**) N2a cells were transfected with GFP/Nedd4 or Nedd4/empty vector using Lipofectamine 3000 reagent (Invitrogen). Cells were then harvested 48 hours post- transfection. Western blotting analysis of Nedd4 and GFP expression. Gapdh was used as a loading control. Quantitative analysis of GFP normalized by Gapdh, respectively. (n = 3, two-tailed student t test, p=0.0629). ns, $p > 0.05$; Data are presented as mean values ± SEM.

(**D**) GFP level is unaffected by Nedd4 ligase activity. N2a cells were transfected with GFP together with wild type Nedd4 (N4) or inactive Nedd4 CS (CS). Samples were collected at indicated time and western blot analysis performed. α-tubulin was used as loading control.

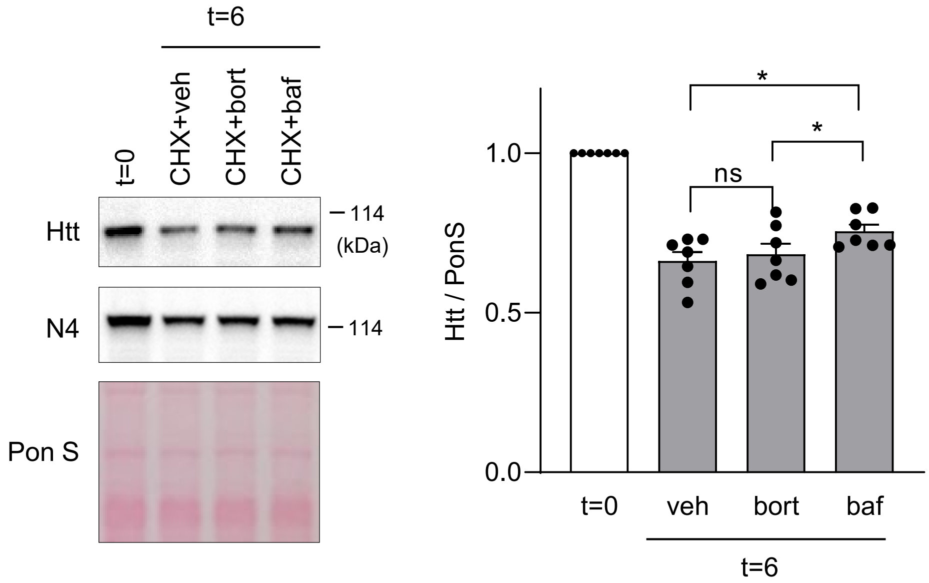

Supplemental Figure 6A. Nedd4-mediated ubiquitination of Htt leads to its preferential degradation by autophagy/lysosomal pathways.

N2a cells were transfected with Htt571-72Q together with Nedd4. Cycloheximide (CHX) chase experiment was performed with 6 h chase with or without treatment of bortezomib (bort) or bafilomycin A1 (baf). Western blot was performed after collecting all the samples. Veh stands for vehicle treatment. Ponseau S (Pon S) was used to ensure even loading. n=7, Tukey's multiple comparison test (*p<0.05, ns, not significant).

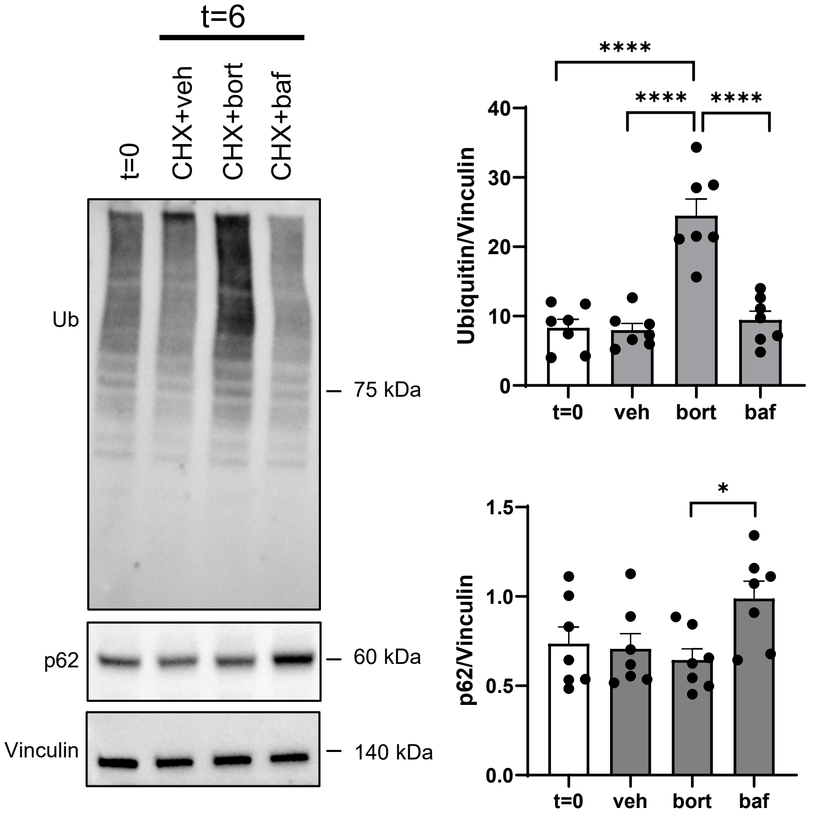

Supplemental Figure 6B. Changes in the levels of ubiquitinated proteins and p62 following chemical treatments.

N2a cells were transfected with Htt571-72Q and Nedd4 by using Lipofectamine 3000 reagent (Invitrogen). 24 hours after transfection, cycloheximide (CHX) chase experiment was performed with 6 h chase, with or without treatment of bortezomib(bort) or bafilomycin A1 (baf). Western blotting was conducted to examine the expression of ubiquitin and p62. Vinculin was used as a loading control (Veh, vehicle control). (Upper graph) Quantitative analysis of ubiquitin normalized by vinculin. (n = 7, one-way ANOVA with Tukey post-tests, t=0 vs bort, $p < 0.0001$; veh vs bort, $p < 0.0001$; bort vs. baf, $p < 0.0001$). (Lower graph) Quantitative analysis of p62 normalized by vinculin. (n = 7, one-way ANOVA with Tukey post-tests, bort vs. baf, $p = 0.0432$). **** $p < 0.0001$; * $p < 0.05$. Data are presented as mean values ± SEM.

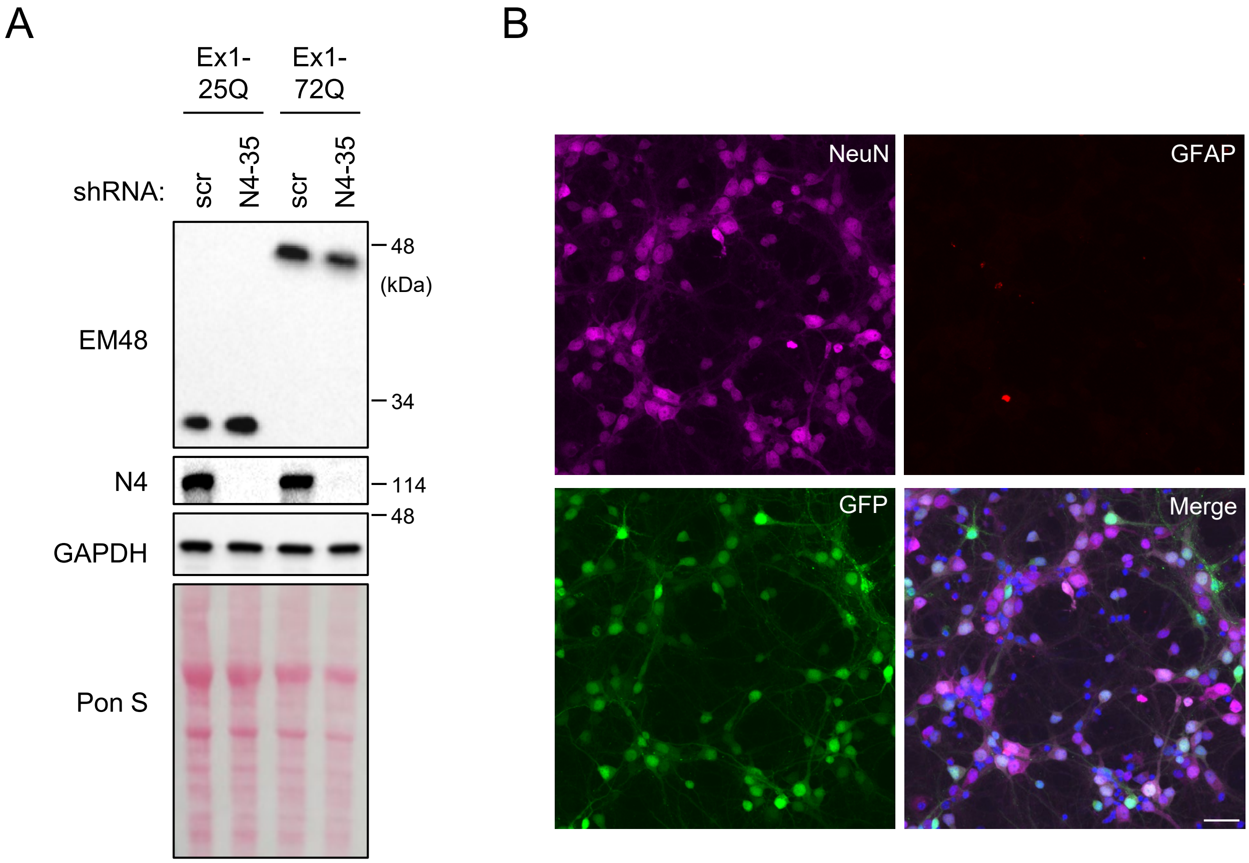

Supplemental Figure 7. Verification of Nedd4 knockdown (KD) in primary cortical neurons.

(**A**) The samples shown in **Figure 3C** were lysed, and western blot analysis was performed using the indicated antibodies. GAPDH was used as a loading control. Western blot analysis demonstrates complete loss of endogenous Nedd4 (N4) by the lentiviral Nedd4-shRNA (N4-35) expression .

(**B**) Double immunostaining of NeuN and GFAP in mouse primary cortical neurons transduced with lentivirus packaged with pLKO.1-U6-shRNA-NEDD4-EF1α-copGFP (scale bar: 100 μm) 24 hours after plating and subsequently cultured for additional six days. Double immunostaining of NeuN and GFAP indicates that our primary neuronal culture is predominantly composed of neurons, and the expression of Nedd4 shRNA, as judged by GFP-signal, was primarily detected in neurons.

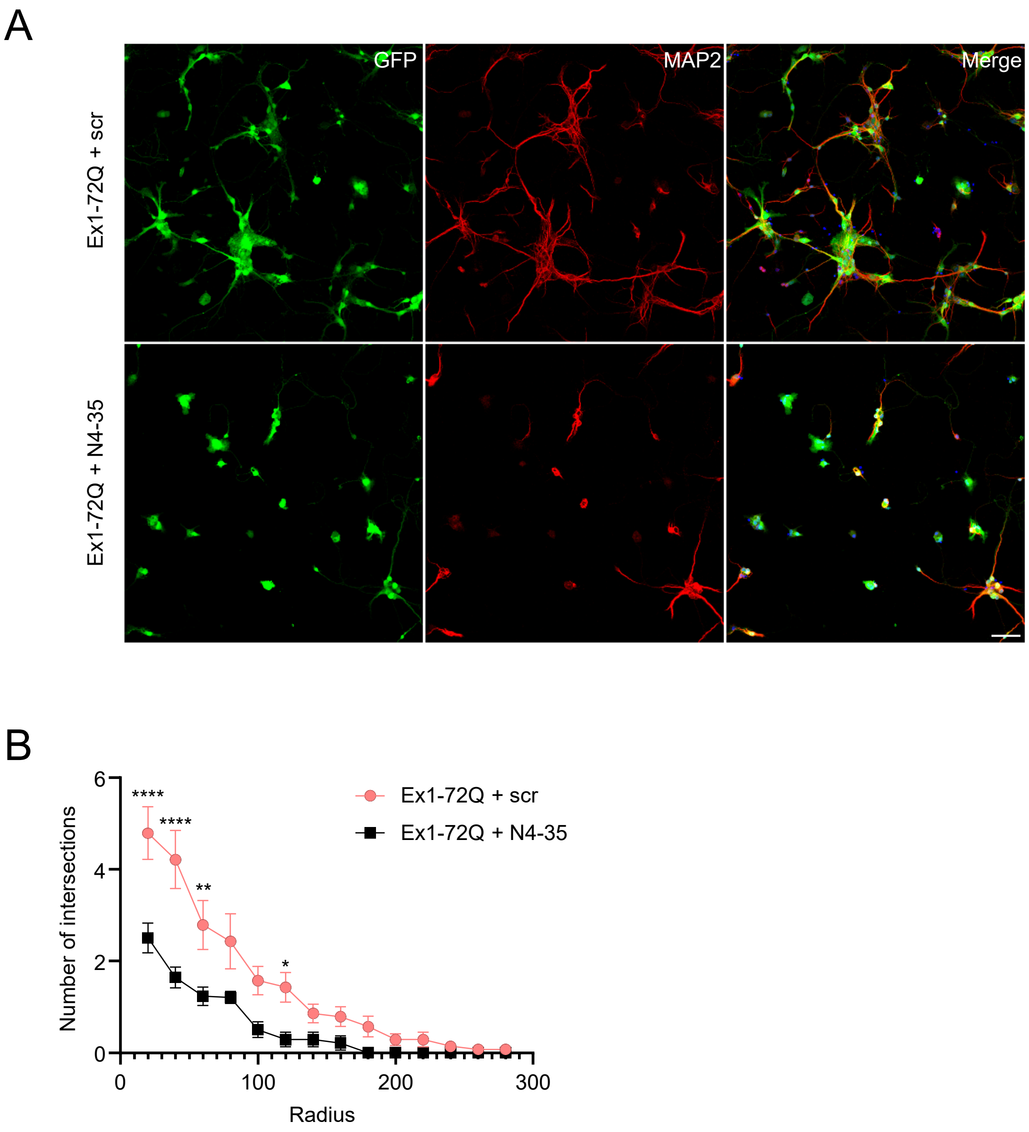

Supplemental Figure 8. The reduction of endogenous Nedd4 exacerbates the neurotoxic effects of mHtt.

(**A**) Neurite reduction in neurons infected with mHTT and Nedd4 shRNA. Mouse primary cortical neurons were transduced with lentivirus expressing Exon1 mHtt and lentivirus expressing scrambled (scr) or shNedd4-35 (N4-35) 24 hours after plating and subsequently cultured for additional six days. Double immunostaining of MAP2 and GFP was used to show neuronal morphology (scale bar: 100 µm).

(**B**) Sholl analysis was conducted to quantify neurite branching in primary cortical neurons ($n = 14$ from three mice, two-way ANOVA, 20 radius, $p < 0.0001$; 40 radius, $p < 0.0001$; 60 radius, $p = 0.0015$; 120 radius, $p = 0.0493$).

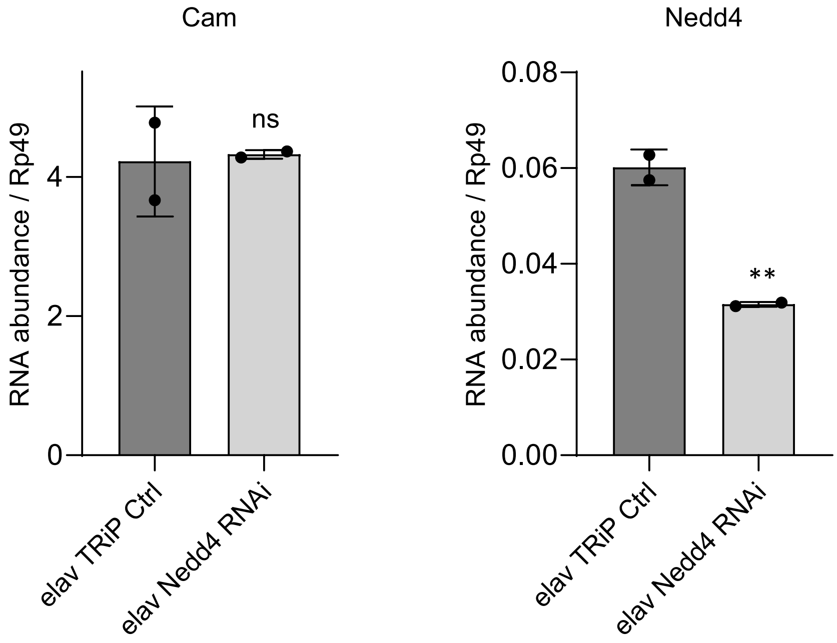

Supplemental Figure 9. **Nedd4 RNAi reduces the Nedd4 RNA levels.**

Heads were collected from flies with a pan-neuronal expression of Ctrl RNAi (elav TRiP Control) or Nedd4 RNAi (elav Nedd4 RNAi) for RNA extraction and qPCR. Relative RNA abundance of Cam (as a control) and Nedd4 normalized to Rp49 is shown (t-test, n=2, **p<0.01, ns, not significant, error bars represent standard deviation).
