# Supplementary material for: Ubiquitin ligase Nedd4 regulates the abundance and toxicity of mutant huntingtin
Source: JCI Insight. 2026 Feb 23;11(4):e181013. doi: 10.1172/jci.insight.181013 (PMC12956003; doi:10.1172/jci.insight.181013)

Figure 1A

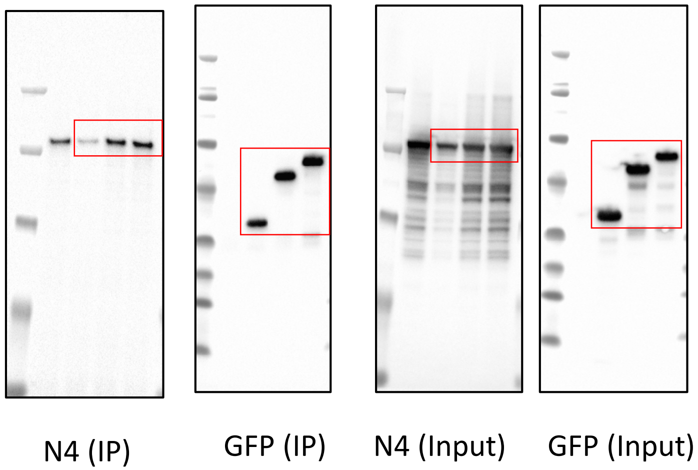

Figure 1B

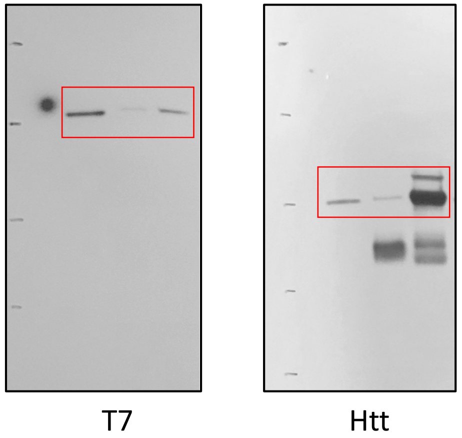

Figure 1C

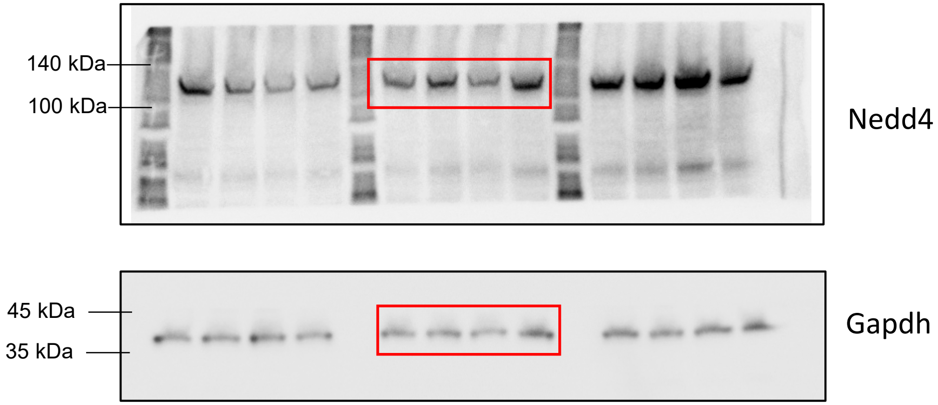

Figure 1E

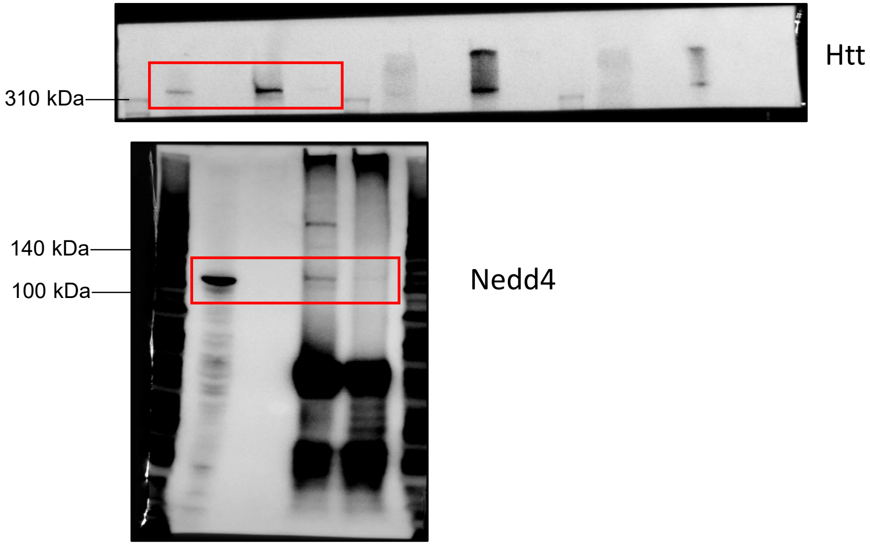

Figure 1F

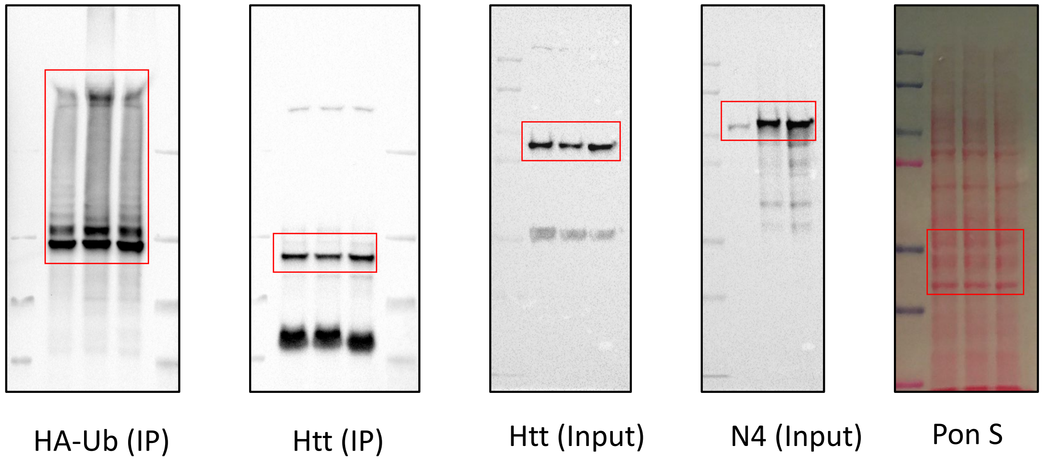

Figure 1G

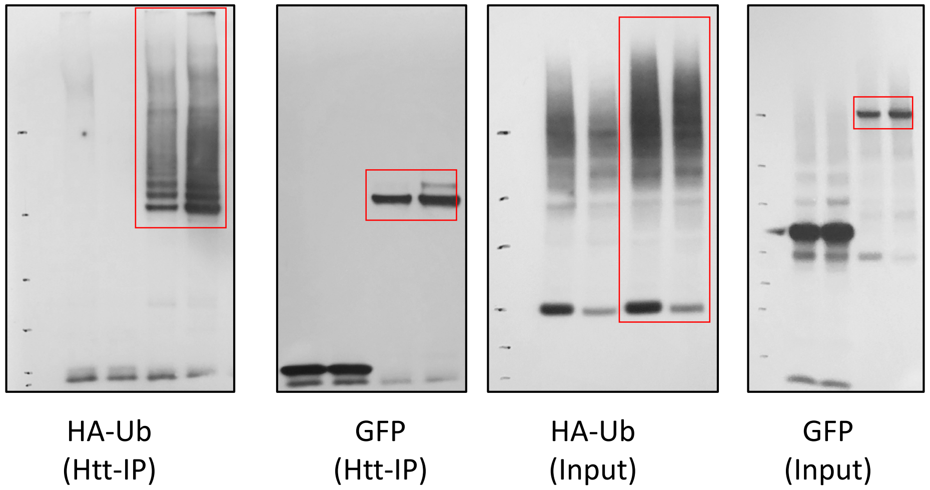

Fig. 2A

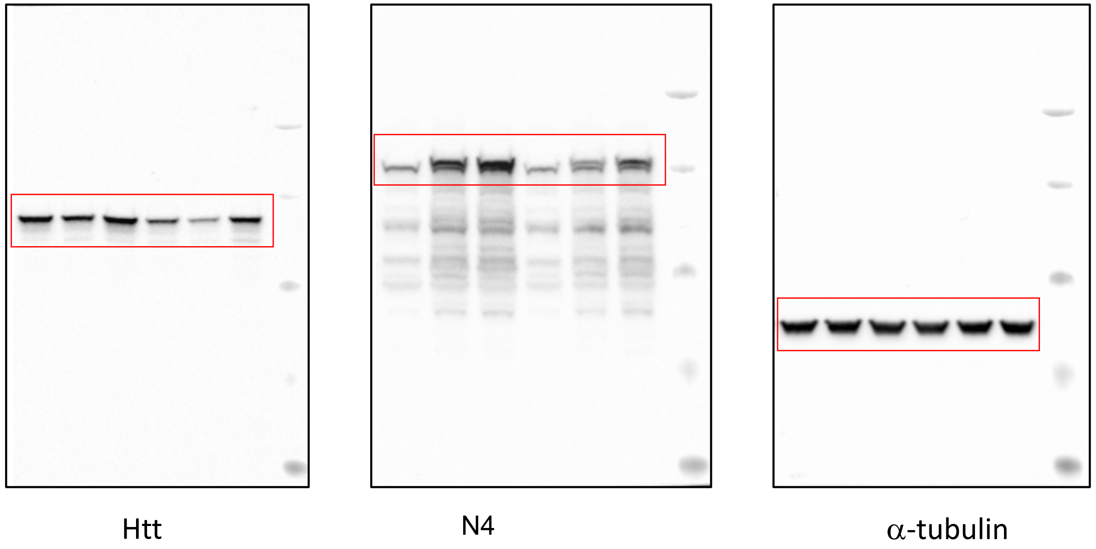

Fig. 2B

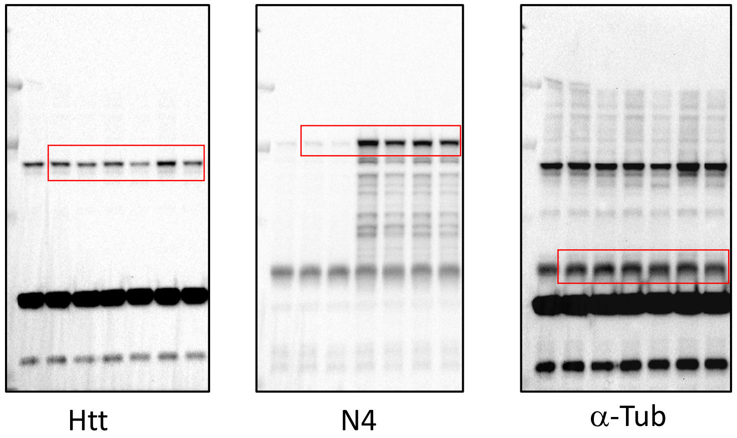

Fig. 2C

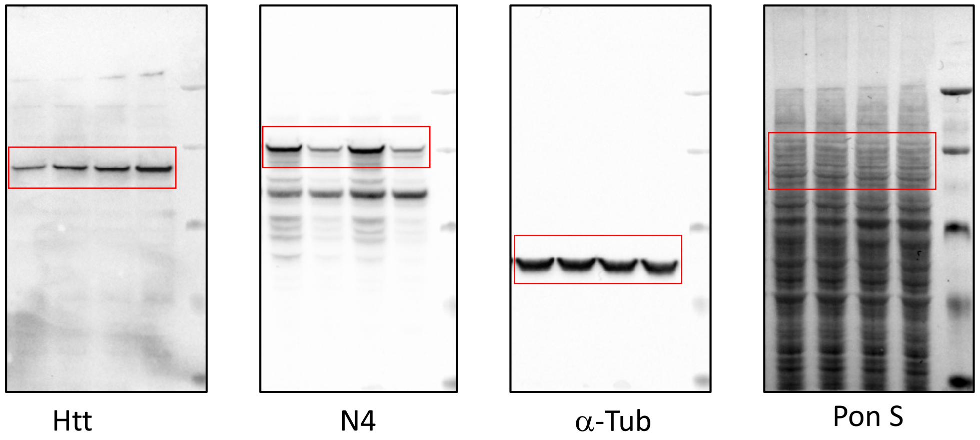

Fig. 2D

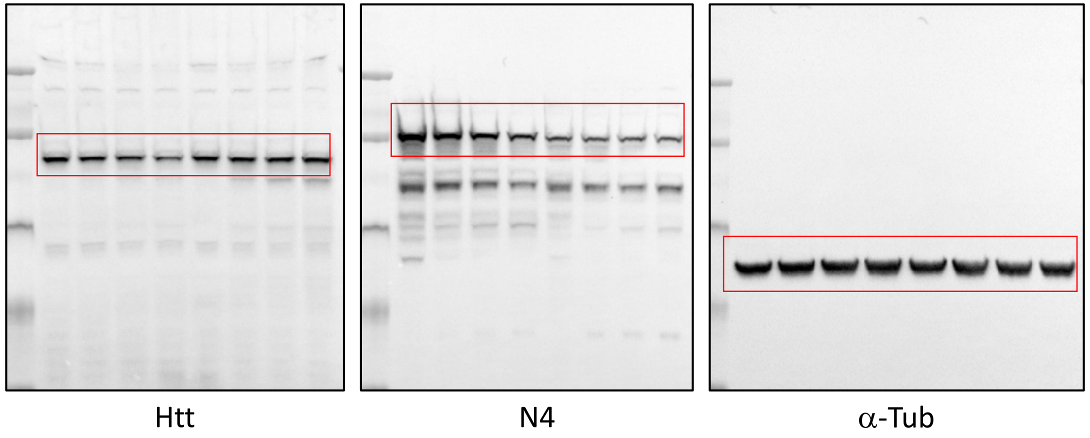

Fig. 2E

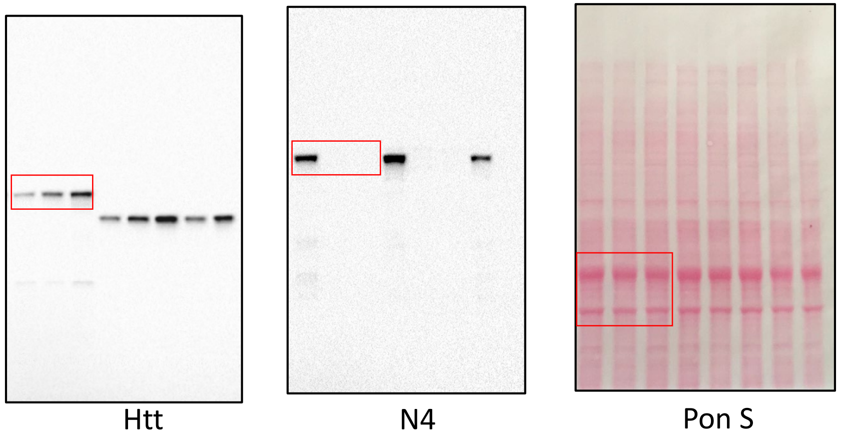

Fig. 5A

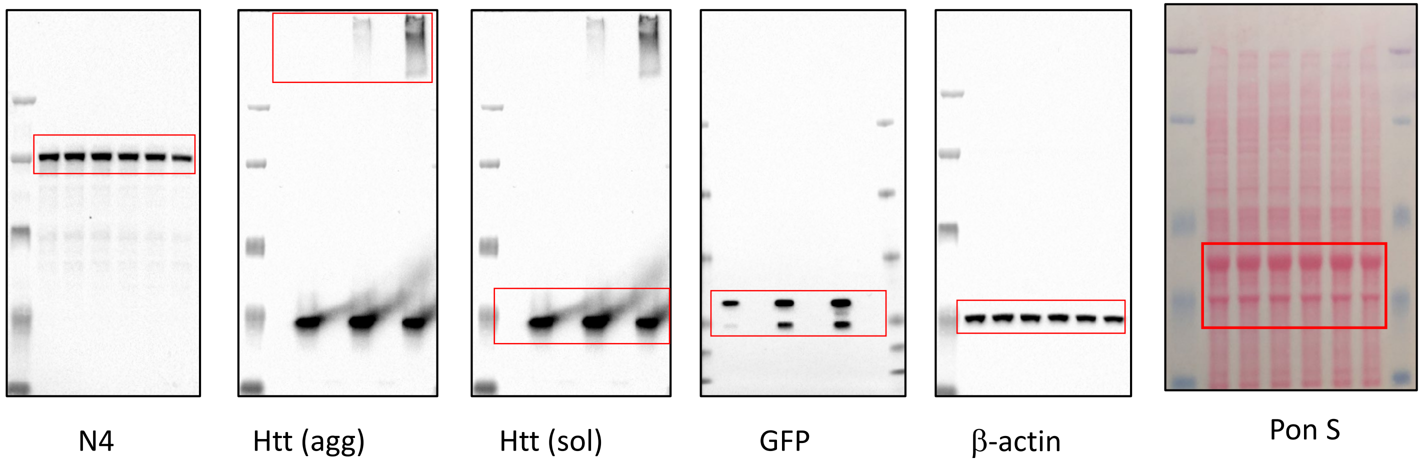

Fig. 5B

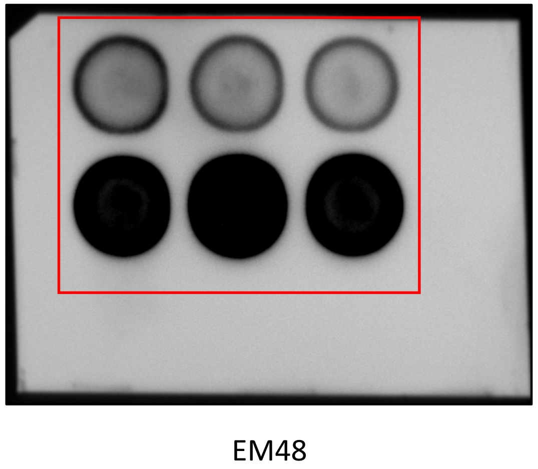

Fig. 5C

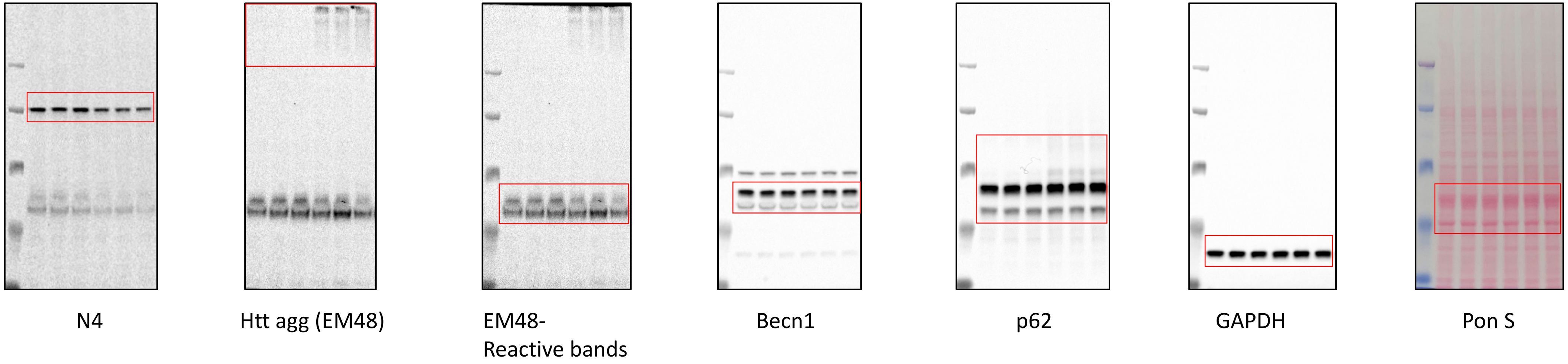

Fig. 5D

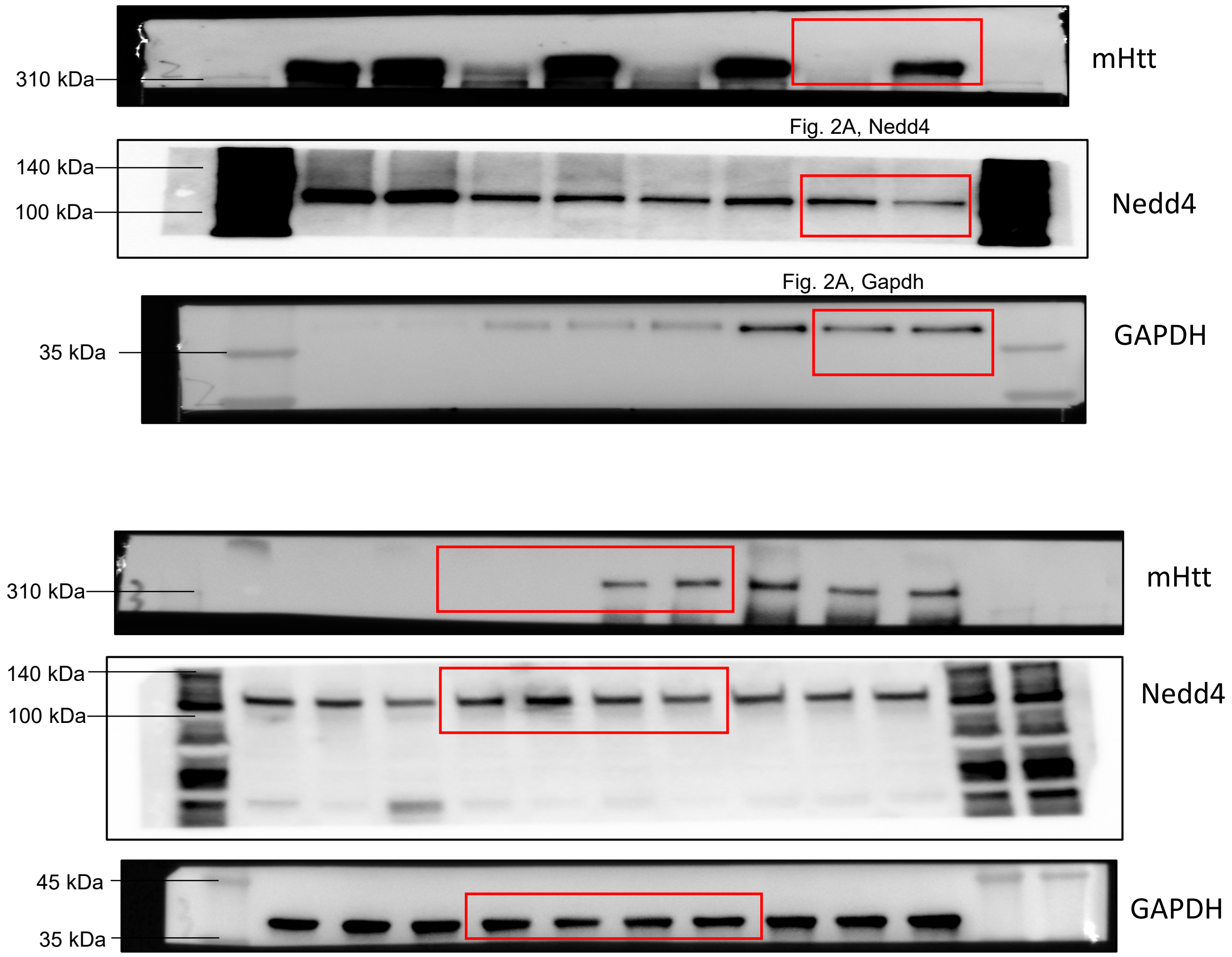

Fig. 5E

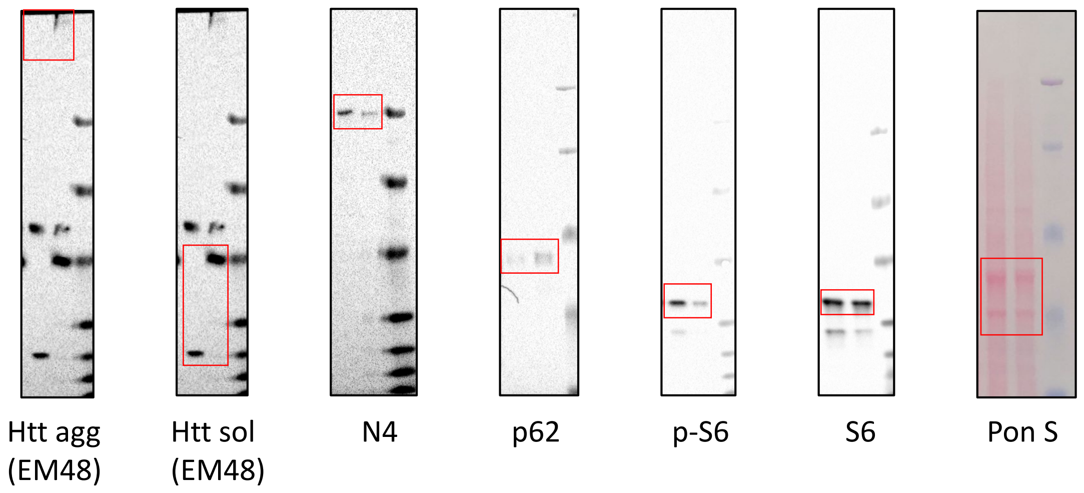

Fig. 5F

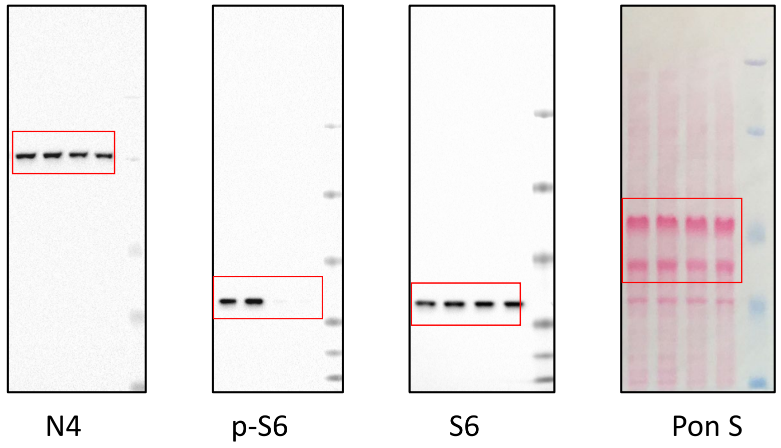

Supp Fig2A

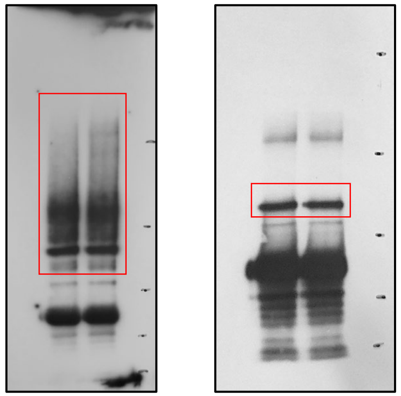

HA-Ub p53-GFP

Supp Fig2B

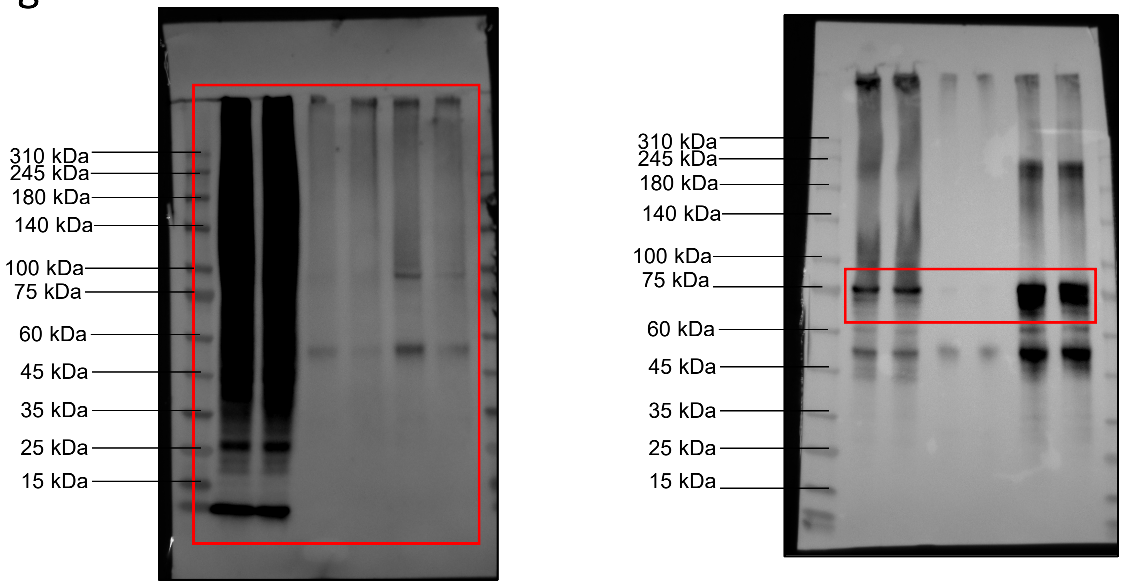

HA-Ub p53-GFP

Supp Fig. 3

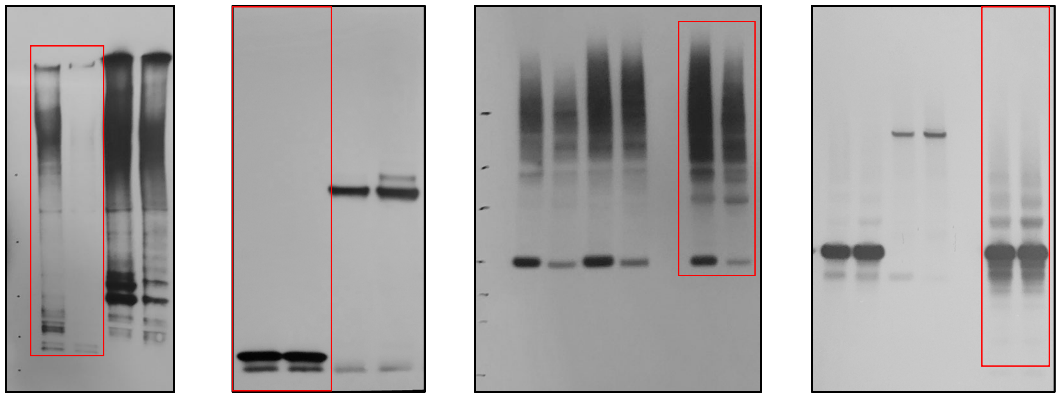

HA-Ub (IP:GFP) | GFP (IP:GFP) | HA-Ub (Input) | GFP (Input)

Supp Fig. 4A

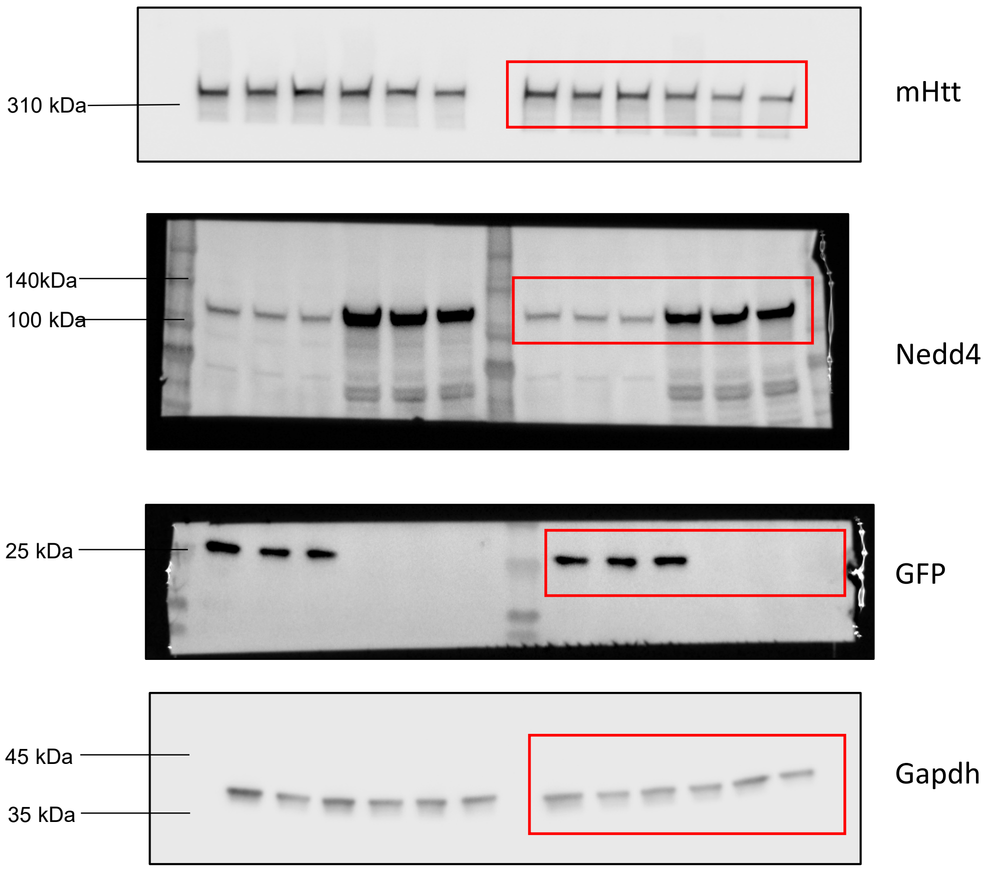

Supp Fig. 4B

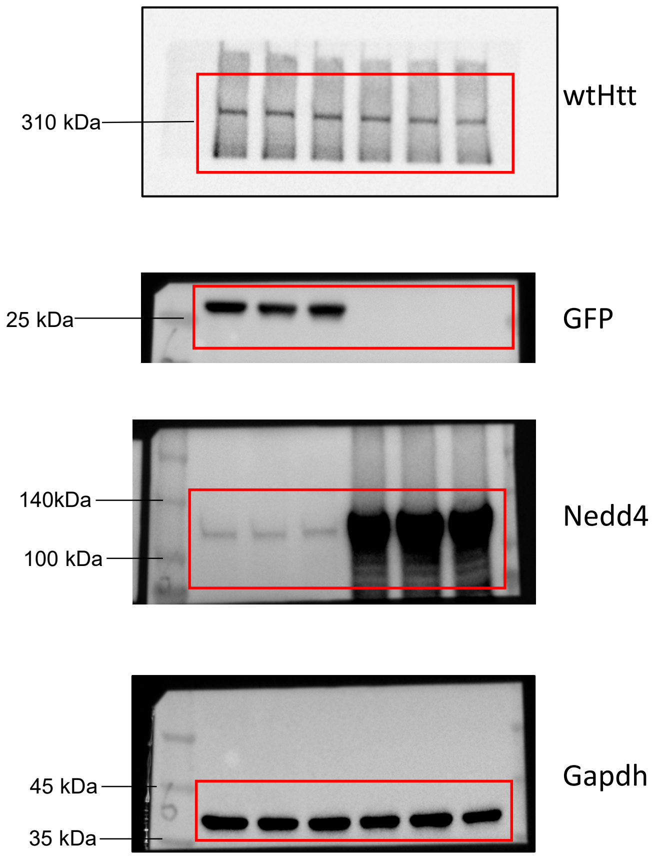

Supp Fig.5A

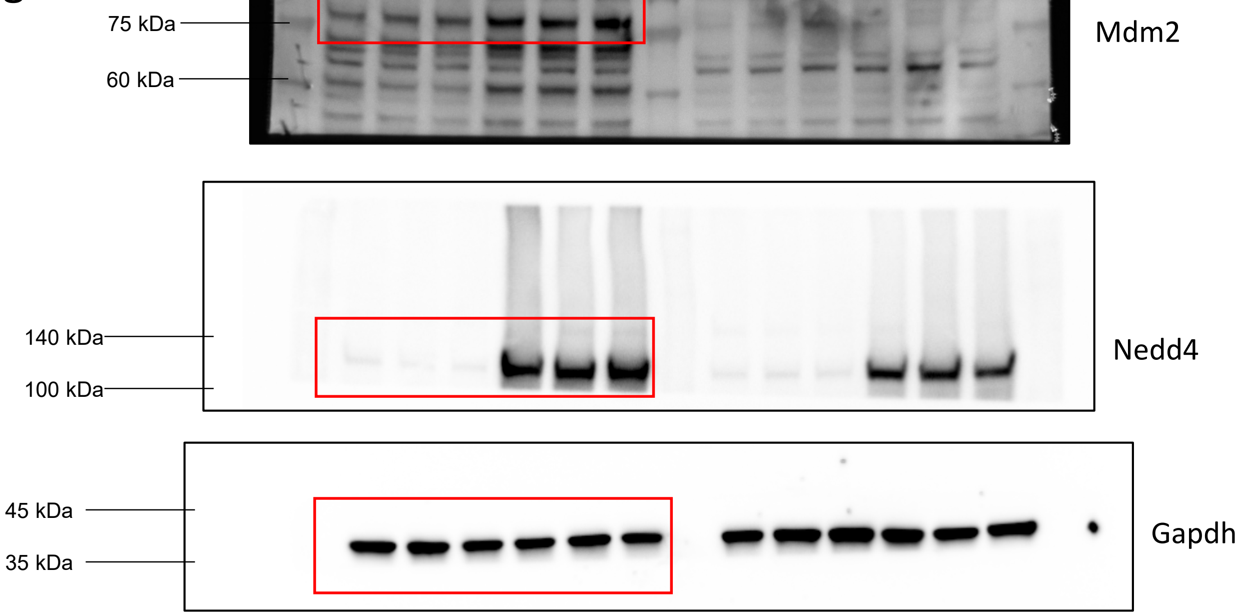

Supp Fig 5B

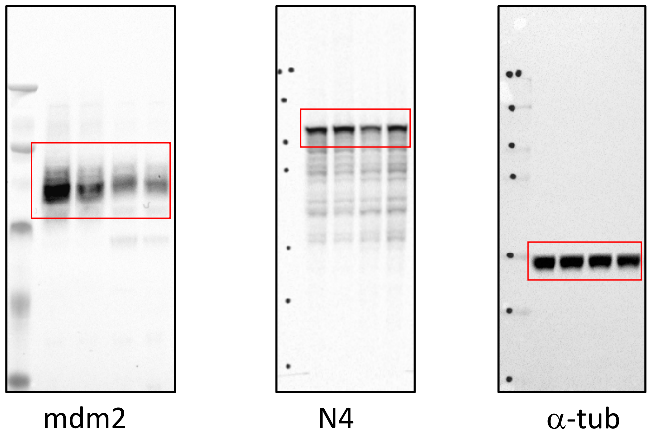

Supp Fig.5C

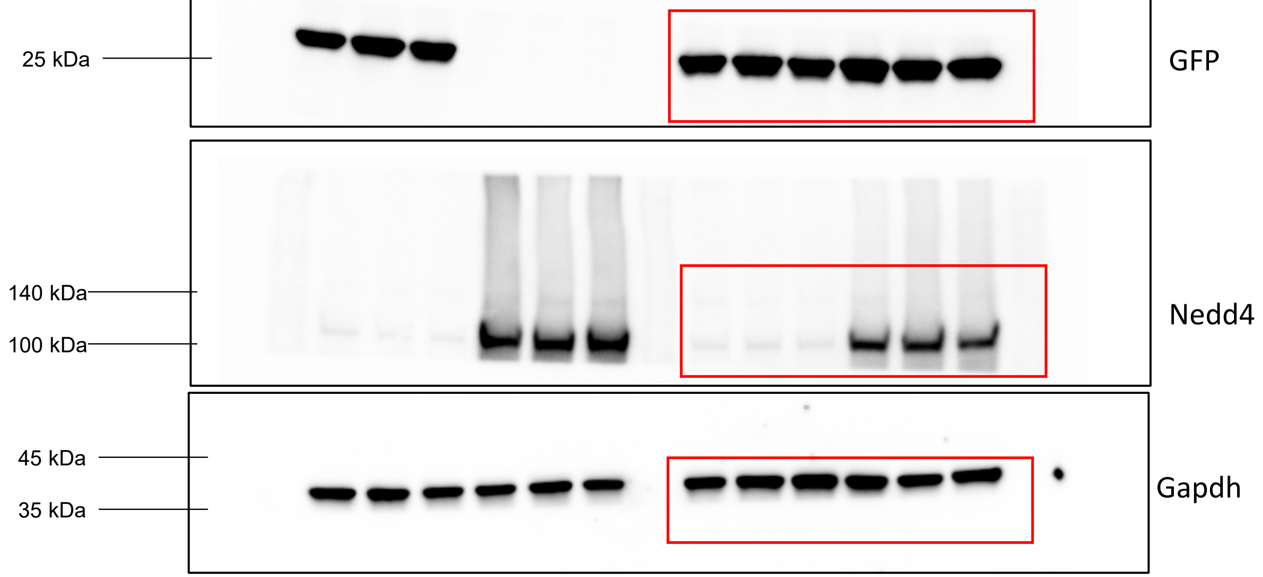

Supp Fig. 5D

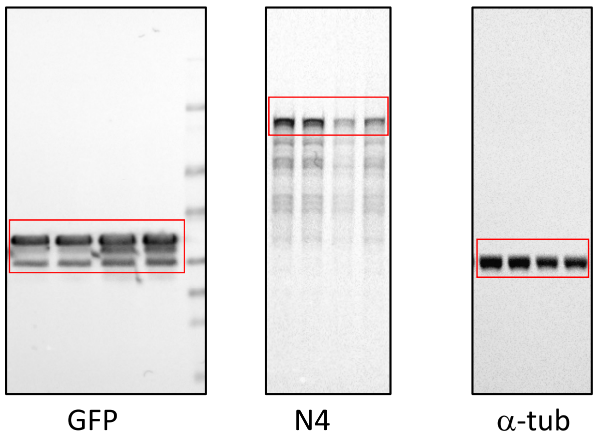

Supp Fig.6A

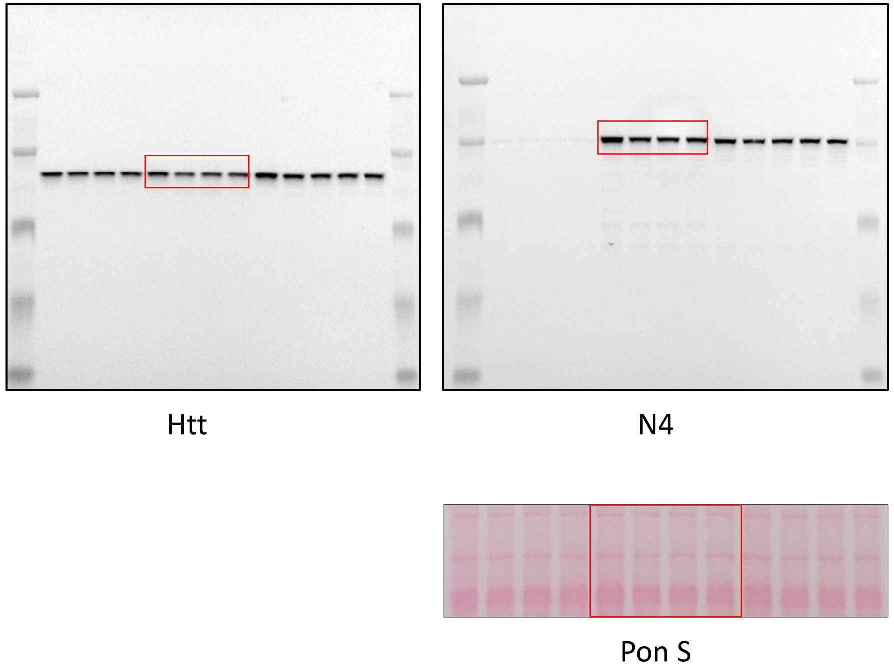

Supp Fig.6B

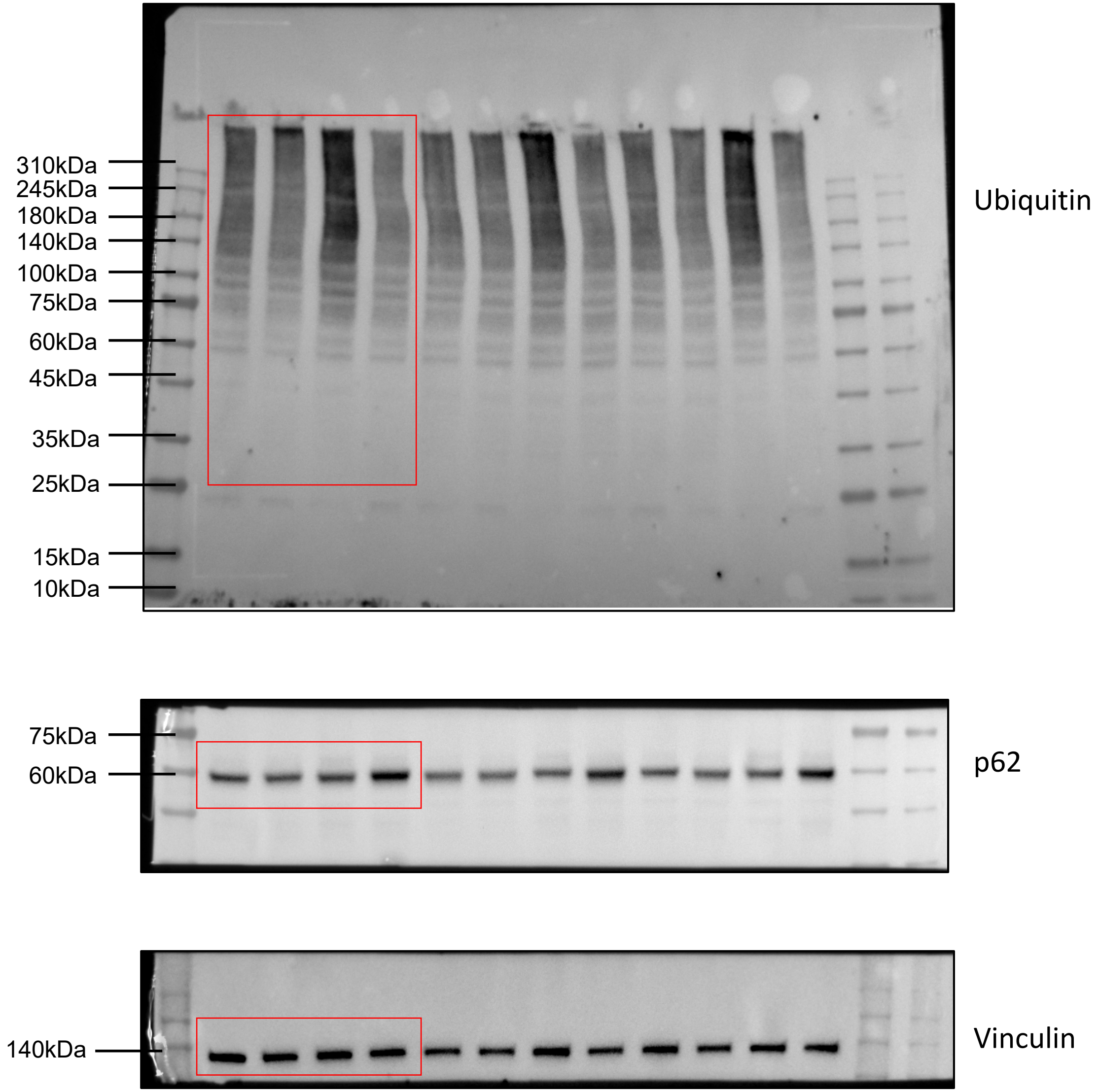

Supp Fig. 7A

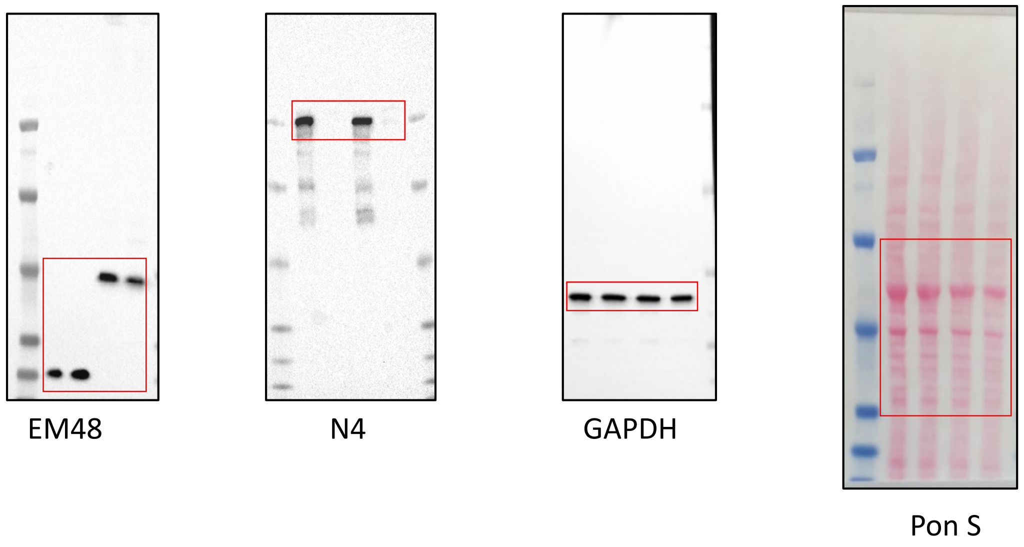

Supplement: Unedited blot and gel images [file jciinsight-11-181013-s046.pdf]
